# Nutrigenomic and Nutritional Analyses Reveal the Effects of Pelleted Feeds on Asian Seabass (*Lates calcarifer*)

**DOI:** 10.1371/journal.pone.0145456

**Published:** 2015-12-22

**Authors:** Si Yan Ngoh, Daniel Tan, Xueyan Shen, Purushothaman Kathiresan, Junhui Jiang, Woei Chang Liew, Natascha May Thevasagayam, Hsiao Yuen Kwan, Jolly M. Saju, Sridatta R. S. Prakki, Chin Heng Goh, Hong Ching Wong, Tai Teck Chan, Miklós Mézes, László Orbán

**Affiliations:** 1 Reproductive Genomics Group, Temasek Life Sciences Laboratory, 1 Research Link, National University of Singapore, Singapore, Singapore; 2 School of Biological Sciences, Nanyang Technological University, 60 Nanyang Drive, Singapore, Singapore; 3 Marine Aquaculture Centre, Agri-Food and Veterinary Authority, St. John’s Island, Singapore; 4 Department of Biological Sciences, National University of Singapore, 14 Science Drive, Singapore, Singapore; 5 Fish Facility, Temasek Life Sciences Laboratory, 1 Research Link, National University of Singapore, Singapore, Singapore; 6 Department of Nutrition, Szent István University, Gödöllő, Páter Károly utca 1, Hungary; 7 Department of Animal Sciences and Breeding, Georgikon Faculty, University of Pannonia, Deák Ferenc u. 16, Keszthely, Hungary; 8 Centre for Comparative Genomics, Murdoch University, 90 South Street, Murdoch, Australia; 9 Department of Aquaculture, Szent István University, Gödöllő, Páter Károly utca 1, Hungary; University of Nordland, NORWAY

## Abstract

As nutrition-related expenses constitute the majority of the costs for aquaculture farms, it is essential for them to use feeds that provide an ideal combination of nutrients for the species of choice. In this study, the relative effect of consuming three different pelleted feeds (B, C and D) in comparison to frozen baitfish (A; control) were compared on juvenile Asian seabass (77.3 ± 22.4g) that were selected for increased growth rate over two generations. Our objectives were: 1) to evaluate the effects of different pelleted feeds based on overall physiological changes and nutritional quality of fillets; 2) improve our understanding of the underlying mechanisms with transcriptomic analysis; 3) if possible, identify the feed type that supports the growth of these fishes without substantially reducing the nutritional quality of fillet. The growth performance, fatty acid composition of fillet, hepatic histology and transcriptome of the fishes (Groups A-D) were analyzed. The majority of fatty acids of the fillets, except γ-linolenic acid (GLA, C18:3n6), correlated significantly with the respective diets. Asian seabass fed Feed C showed highest specific growth rate (SGR) and feed conversion efficiency (FCE) with closest histology and transcriptomic profile to control, but their fillet contained the highest n6/n3 ratio. When the liver-based transcriptomes were analyzed, a complex set of differentially expressed genes were detected between groups fed pelleted feeds and controls as well as among the pellet-fed groups themselves. Significant enrichment of genes with growth-related function tallied with the morphological data measured. When compared with control (Group A), ‘Biosynthesis of unsaturated fatty acids’ and ‘Steroid biosynthesis’ pathways were significantly enriched in pellet-fed groups. Reduced goblet cell numbers were observed in the gut of pellet-fed fish compared to controls and *fads6* was found to be a suitable candidate gene to separate wild-caught Asian seabass, from pellet-fed ones. These results provide insights for researchers on the various effects of feeds on the biochemistry and global gene expression of the fish and potentially for seabass farms to make more informed feed choices.

## Introduction

The global aquaculture production of food fish has increased tremendously over the last decade, reaching 62.7 million metric tonnes in 2011 or about 40.1% of world total fish production [1]. Asian seabass *(Lates calcarifer)* or barramundi is an important aquaculture species native to the Indo-West Pacific region [2] with increasing production currently estimated at 67,000 tonnes [3]. In collaboration with the Marine Aquaculture Centre of Agri-Food and Veterinary Authority (MAC, AVA, Singapore), we have been performing a selection program to develop elite lines of Asian seabass since 2004 through the utilisation of molecular genetics and genomic tools, such as genotyping, linkage mapping and transcriptomics [4–6]. At the beginning, we have focused onto increasing the growth potential and achieved a substantial increase in the growth rates in both the F1 and F2 generations. Recently, we have started to work on improved disease resistance as well and we intend to improve our understanding on the effects of feeds onto the physiology of the seabass using genomic tools.

Asian seabass is known to spawn primarily in seawater, spends the first 1–2 years of its life in freshwater and then migrates back to seawater for breeding [7]. The catadromous nature of the species has motivated numerous studies to investigate its fatty acid metabolism [8–10]. In marine fishes, the ability to convert eicosapentaenoic (EPA, C20:5n3) to docosahexaenoic acids (DHA, C22:6n3) might be possible but the conversion rate is usually too low to meet the high demand for DHA in rapidly growing and developing fry and fingerlings [11]. The fatty acid biosynthetic capability of Asian seabass is usually considered to be similar to other marine teleosts. As such, it would require feeds that contain much higher levels of fish oil as it does not possess or has only limited ability to convert C18 polyunsaturated fatty acids (PUFA) into long-chain PUFA (lcPUFA) [12,13]. It is well established that fish oil- and fish meal-based feeds are essential for Asian seabass, as their absence would result in growth retardation and reduction in all major n3-lcPUFAs, including EPA and DHA [8,9].

In addition to studies performed to analyze the fatty acid requirements of Asian seabass, several research groups have also evaluated their growth performance by varying carbohydrate and lipid inclusion levels [14,15], optimised dietary protein and energy ratios [16,17] and determined the requirements of certain vitamins and minerals [18–22]. Others focused on finding suitable alternative sources of feed ingredients derived from plant, animal or microbial origin to replace fish meal and fish oil due to their scarcity and increasing prices [23–25]. Despite those efforts, the nutritional information required to formulate precision diets for Asian seabass is still incomplete. To date, different feed manufacturers have attempted to produce complete feeds that fulfil the nutrient requirements of various cultured species by using different ingredients. However, no clear consensus seems to have been established on the needs of the species. As a result, a diverse range of feeds compounded with different proportions of ingredients being offered and used for its culture. According to our knowledge, no one has analysed the potential effect of multiple pelleted grow-out feeds on various performance parameters and fillet fatty acid composition of the Asian seabass in comparison to frozen baitfish controls.

Recent data has shown that Asian seabass is a species complex containing three genetically distinct varieties [26–28]. As most nutritional studies were performed on individuals from the Australian clade whereas our fishes belong to the South-East Asian one, it was important to analyze the effects of feeds on our selected seabass and identify a feed that will suit their needs with limited compromise regarding the nutritional quality of the fillet thereby preventing subpar utilization of gains achieved through the selection process. Therefore, in our present study, three commercially available pelleted grow-out feeds (Feed B-D) and frozen baitfish (Feed A as control) were evaluated based on the overall growth performance, nutritional quality of fillets and histological changes of the mid-gut and liver in juvenile Asian seabass. These data allow us to glimpse into the effects of different feeds onto the gene expression profile of the liver and with the recent availability of the Asian seabass transcriptome [29], its draft genome [30] and a comprehensive Asian seabass genome assembly [31] more detailed analysis of such datasets will be possible in the near future.

## Materials and Methods

### 2.1 Ethics statement

This study and all experimental procedures using animals were approved by Agri-Food and Veterinary Authority of Singapore Institutional Animal Care and Use Committee (approval ID: AVA-MAC-2012-02). All animal handling protocols comply with guidelines set by the National Advisory Committee on Laboratory Animal Research (NACLAR) for the care and use of animals for scientific purposes in Singapore. All animals were sacrificed or euthanized by overdose of Tricaine methane-sulfonate (MS-222; at least 300 mg/L for over 10 minutes until total loss of gill movement) following AVMA (American Veterinary Medical Association) guideline for euthanasia of animals.

### 2.2 Origin, breeding and culture of experimental animals

In order to reduce the effect of genetic variability on the results observed, fish used for the study were spawned from a single pairwise cross between two of our selected F1 Asian seabass brooders with increased growth rate. Prior to crossing, the parents were genotyped with nine polymorphic microsatellites [32] and results showed that they were 42.5% similar. Spawning was synchronized through hormonal induction with Luteinizing Hormone Releasing Hormone A (LHRHa, Argent Labs, Redmond, USA) according to the lunar cycle.

Fertilized eggs were collected and incubated in well-oxygenated seawater at 28–29°C. Upon hatching, the larvae were stocked in a flow through fibreglass tank at a density of 20 larvae per litre of filtered seawater between 2 to 18 days post-hatch (dph), larvae were reared on live feed. Weaning of live feed to dry commercial feed was performed with Otohime B1 (Marubeni Nisshin, Japan) from 18 to 21 dph, followed by Otohime B2 between 22 to 25 dph. Grading was performed weekly from 25 to 60 dph to minimize cannibalism. Fry were fed commercial compound feeds of increasing pellet size: BioMar INICO Plus (Nersac, France; 0.8–1.9 mm) from 26 dph to 59 dph and BioMar Efico YM 668 (3–4.5mm) from 60 dph to 103 dph.

### 2.3 Feeding trial

**Fig 1:**
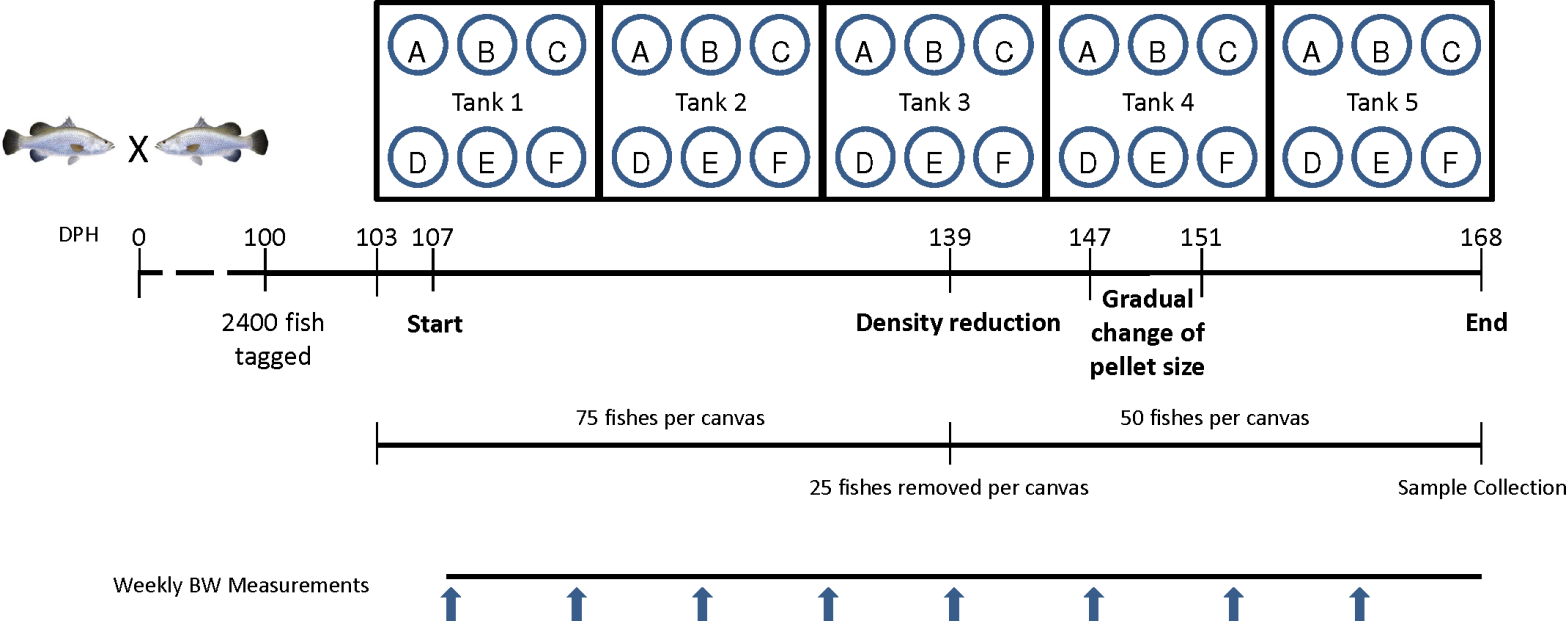
Schematic overview of the experimental design. Five large tanks containing six individual canvases of two metric ton volume each per tank were set up. Asian seabass siblings (103 dph) from a pairwise cross performed with two brooders selected for increased growth rate were tagged, measured, photographed and placed into the canvases at 75 individuals per canvas initial density. Following a short adaptation period, frozen baitfish (Feed A, control) and five commercial pelleted growout feeds (Feed B-F) were fed to their respective groups at an average body weight (BW)/day for 61 days; Group A: 6.1%; Group B, C, D, E: 2.2%; Group F: 1.8%. The amount of feed was adjusted on a weekly basis based on BW measurements of 15 individuals per canvas. At 139 dph, all the fish were measured and 25 individuals were randomly removed to reduce density. During the 147–151 dph period, gradual change in pellet size fed to fishes in groups B, C, D and F. At the end of the experiment all individuals were measured and photographed and samples were collected for the different analyses. [Only fish from Groups A-D were subjected to detailed analysis, whereas the other two groups (E&F) were excluded due to technical reasons.]

Five 20 m^3^ tanks were set up with six 2m^3^ rectangular canvases suspended into the water of each tank. At 100 dph of age, 2,400 fishes were tagged individually with a passive integrated transmitter tag (8mm PIT Tag, Green Tag Pte Ltd, Singapore). Seventy-five tagged fishes (average weight: 77.3+/-1.6 g) were randomly stocked into each canvas (30 groups in total) starting at a stocking density of 3 kg/m^3^ (that increased to 18.75 kg/m^3^ by the end of the trial; **Fig 1**). Variable conditions, including flow rate and temperature, were recorded daily (data not shown). From 103 dph onwards, we started the feeding trial: Frozen baitfish (Feed A; control) and five commercial grow-out pelleted feeds (Feeds B-F) were fed to groups of fishes reared in six separate canvases within each of the five tanks, resulting in five replicate groups for each feed type. Other than the nutritional information provided on the packaging label (**S1 Table**), no other additional information pertaining to the ingredient compositions of the feeds was available. These slow-sinking marine fish feeds were chosen for this feeding trial as they were either currently known to be used for culturing Asian seabass by farmers and/or they were feeds recommended by feed manufactures for the same purpose. The initial pellet sizes used at the start of the experiment ranged from 4.5 to 6.5 mm in diameter (**Table 1**). This was unavoidable due to limitation in the availability of various feed sizes from different manufacturers. The frozen baitfish fish used was a wild-caught mix, comprising different species of the scads (Genus *Decapterus*) with mackerel scad (*Decapterus macarellus*) being in the majority. The three commercial pelleted feeds that were eventually evaluated were as follows: Marine Fish Feed (Gold Coin SDN BHD, Sarawak, Malaysia), Tomboy Skretting BS10 (Skretting, Ho Chi Minh City, Vietnam) and Grouper Feed (Chin Da Enterprise Co., Ltd., PingTung, Taiwan). Arbituary codes (Feeds B, C and D) were assigned randomly to these pelleted feeds and the codes were not linked to any specific manufacturers.

**Table 1:** Measured nutrient content of frozen fish (Feed A, control) and three pelleted feeds (Feeds B-D; two sizes for each type).

Proximate nutrient composition*	A	Bs^†^	Bb^‡^	Cs^†^	Cb^‡^	Ds^†^	Db^‡^
Pellet size (mm)	n.a.	5.0	9.0	5.0	8.0	6.0	9.5
ME (MJ/kg)	13.39	15.42	15.63	14.99	14.85	14.26	14.19
Dry matter (%)	25.16	94.27	91.75	92.17	93.10	92.84	89.91
Crude protein (%)	18.6	48.40	46.53	45.44	46.08	44.91	41.81
Crude fat (%)	2.46	18.10	17.70	19.90	18.30	13.00	12.90
Crude fibre (%)	n.d.	2.22	1.07	2.16	1.58	1.73	2.07
Crude carbohydrate (%)	n.d.	11.05	13.71	8.78	10.33	16.03	14.32
Crude ash (%)	n.d.	9.85	10.00	14.00	13.94	11.86	11.61
Lysine (%)	n.d.	2.75	2.87	2.95	2.88	3.11	2.42
Methionine (%)	n.d.	1.36	1.32	1.10	1.14	0.78	0.65
Cysteine (%)	n.d.	0.76	0.66	0.64	0.67	0.50	0.43

*, Nutrient content is given on wet matter basis.

†, Small pellet size was fed from 107dph to 152dph.

‡, Big pellet size was fed from 147dph to 168dph.

Metabolisable Energy (ME) was calculated according to the equation: ME (MJ/kg dry matter) = -3.064 + 34.82*X_1_ + 17.21*X_2_ + (18.52–31.2*X_4_)*X_3_ where; X_1_ = crude fat (g/g d.m.); X_2_ = crude protein (g/g d.m.); X_3_ = crude carbohydrate (g/g d.m.); X_4_ = crude fibre (g/g d.m.) [83].

n.d. = not determined.

n.a. = not applicable.

Prior to the start of the experiment we have tested the daily feed consumption of the fish by feeding them ad libitum for five days. The amount of feed fed from 107 dph onwards was set slightly below this value to ensure that all the feed fed was consumed. Fish were fed three times a day in small proportions over the period of 30–45 minutes. Feeding was terminated once fish let pellets sunk to the bottom. Due to the fact that canvas tanks had a depth of over two meters, leftover feed, typically a few pellets only, were not collected from the bottom. On a daily average, all the groups on commercial grow-out feeds, except those on Feed F, received 2.2% of their body weight (BW). For Feed F, the amount had to be reduced to 1.8% of BW, as this was the maximum amount the fish were willing to consume, which resulted in about 10% lower daily energy intake as compared to other feeds (data not shown). Due to this reason, Group F was eventually removed from any subsequent analysis. Feed A was fed at an increased percentage of 6.1% of BW daily to maintain the same average energy intake, as the dry matter content of frozen baitfish is substantially lower than those of the commercial feeds (**Table 1**).

The amount fed was adjusted to the growth of the fish at the beginning of every week by measuring the BW of 15 random individuals per canvas. Growth increase was recorded and total BW for each canvas was determined weekly. As this weekly weight measurement disrupted the daily routine of the fishes and they refused to eat, feeding was ceased every Monday. Between 147 to 151 dph, gradual change to larger sized pellet was performed for the groups fed commercial feeds, with the exception of Feed E, where only a single size (4.5 mm) was available. Therefore, Group E was also removed from any subsequent analysis. During the change, on the first day, 20% of the feed was replaced with the larger sized pelleted feed. This was gradually increased to 40%, 60% and 80% on a daily basis and from 151 dph onwards only the larger sized pelleted feed was fed to the fishes. Throughout the feeding trial, a total of 20 individuals from all the groups were lost and they were replaced from a backup group of the same origin that was kept for this purpose. However, these replacement individuals were used only for maintaining the density of the groups and they were excluded during sample collections and any subsequent statistical calculation.

### 2.4 Nutrient content analysis of feeds

As indicated above, five commercial pelleted feeds were used for the feeding trial, but two had to be excluded from the study due to technical reasons. The frozen baitfish control (Feed A) and the remaining three (Feeds B-D) with varying amount of nutrients (**Table 1**) and distinct differences in fatty acid composition (**Table 2**) were analysed in detail in this experiment. The nutrient content of the experimental diets (**Table 1**) were determined using standard techniques for proximate analyses by Food Analytica Ltd. (Gyula, Hungary; accreditation number: NAT-1-1582/2009). Dry matter and ash content of diets were determined by heating at 105°C until stable weight and at 500°C in a muffle furnace, respectively. Crude protein was determined using a Kjeltec auto system (Tecator, Hoganàs, Sweden). Crude fat was determined using the standard ether extraction in a Soxhlet apparatus. Starch was quantified as glucose after starch hydrolysis with a heat tolerant amyloglucosidase [33]. Amino acids were analysed by HPLC method with post-column derivatisation as described by the Commission Directive of the European community [34]. Fat was extracted for fatty acid analysis using the methods of Folch and colleagues [35]. Fatty acid methyl esters (FAME) were derivatised using the procedure as described by Christie [36] and analysed by gas liquid chromatography performed on Shimadzu 2100 apparatus (Shimadzu, Kyoto, Japan), equipped with a SP-2380 capillary column (Supelco, Bellefonte, USA). To identify individual FAMEs in the chromatogram, a FAME standard mixture (Me100; Larodan Fine Chemicals, Malmö, Sweden) was used. Results were expressed as area % of the total FAME (**Table 2**).

**Table 2:** Fatty acid composition of frozen fish (Feed A) and three pelleted feeds (Feeds B-D; two sizes for each type).

Fatty acid composition (Area %)	Feeds						
	A	Bs^†^	Bb^‡^	Cs^†^	Cb^‡^	Ds^†^	Db^‡^
Caprylic (C8:0)	0.47	0.05	0.21	0.08	0.10	0.05	0.06
Capric (C10:0)	0.78	0.07	0.20	0.08	0.00	0.00	0.00
Lauric (C12:0)	1.11	0.14	0.31	0.16	0.09	0.21	0.25
Tridecanoic (C13:0)	0.00	0.00	0.00	0.00	0.00	0.00	0.07
Myristic (C14:0)	8.70	6.82	6.86	2.56	2.48	9.29	7.58
Pentadecanoic (C15:0)	1.48	0.43	0.49	0.21	0.21	0.78	0.62
Palmitic (C16:0)	28.39	19.32	20.70	19.78	20.08	26.33	27.53
Heptadecanoic (C17:0)	1.50	1.00	0.34	0.26	0.24	0.73	0.59
Stearic (C18:0)	10.46	4.01	4.66	6.05	5.53	4.35	4.38
Arachidinic (C20:0)	0.15	0.03	0.46	0.28	0.23	0.44	0.27
Henecosaenoic (C21:0)	0.00	0.04	0.15	0.00	0.00	0.08	0.02
Behenic (C22:0)	1.45	0.00	0.12	0.10	0.00	0.23	0.34
Tricosanoic (C23:0)	0.19	0.49	0.00	0.00	0.00	0.34	0.28
Lignoceric (C24:0)	1.00	1.40	1.34	0.36	0.37	0.82	0.80
ΣSFA	55.68	33.80	35.84	29.92	29.33	43.65	42.79
Myristoleic (C14:1)	0.27	0.13	0.15	0.06	0.07	0.04	0.17
cis-10 Pentadecanoid (C15:1)	0.10	0.00	0.00	0.00	0.00	0.06	0.06
Palmitoleic (C16:1)	6.78	7.23	7.20	3.76	3.87	9.06	7.41
cis-10 Heptadecanoic (C17:1)	0.34	1.32	1.16	0.35	0.18	1.55	1.07
Oleic (C18:1n9c)	4.76	14.90	14.35	35.27	33.73	10.39	15.46
cis-11 Eicosenoic (C20:1)	0.24	0.00	0.18	0.74	0.70	0.00	0.11
ΣMUFA	12.49	23.58	23.04	40.18	38.55	21.10	24.28
Linoleic (C18:2n6)	1.44	11.52	9.90	18.31	19.85	7.02	8.52
γ-Linolenic (C18:3n6)	0.21	0.24	0.23	0.11	0.11	0.30	0.23
cis-11,14 Eicosadienoic (C20:2 n6)	0.15	0.00	0.00	0.23	0.17	0.00	0.11
cis-8,11,14 Eicosatrienoic (C20:3 n6)	0.00	0.12	0.12	0.31	0.07	0.00	0.11
Arachidonic (C20:4n6)	1.96	0.75	0.80	0.28	0.33	1.24	1.09
Σn6-PUFA	3.76	12.63	11.05	19.24	20.53	8.56	10.06
α-Linolenic (C18:3n3)	0.70	1.37	1.13	2.55	2.74	0.91	0.83
cis-11,14,17 Eicosatrienoic (C20:3 n3)	0.00	0.00	0.23	0.04	0.00	0.00	0.00
cis-5,8,11,14,17 Eicosapentaenoic (C20:5n3)	7.93	13.51	13.61	2.26	2.58	9.61	8.35
cis-4,7,10,13,16,19 Docosahexaenoic (C22:6n3)	9.49	5.01	5.90	1.90	1.96	6.72	6.42
Σn3-PUFA	18.12	19.89	20.87	6.75	7.28	17.24	15.60
ΣPUFA	21.88	32.52	31.92	25.99	27.81	25.80	25.66
Σn6/Σn3 PUFA	0.21	0.63	0.53	2.85	2.82	0.50	0.64
DHA/EPA	0.52	0.37	0.43	0.84	0.76	0.70	0.77

†, Small pellet size was fed from 107dph to 152dph.

‡, Big pellet size was fed from 147dph to 168dph.

Mean values are represented in area % of total fatty acids, SFA = Saturated fatty acids; MUFA = Monounsaturated fatty acid; PUFA = Poly unsaturated fatty acid.

### 2.5 Measurements, quantification of traits and sampling

At the beginning, pictures were taken from 2,250 individuals and they were placed into the canvases (the remaining 150 tagged individuals were kept as backup to replace lost individuals during the period of the experiment). The fish were sedated with 31mg/L of AQUI-S^®^ (AQUI-S New Zealand Ltd., Lower Hutt, New Zealand) for 10 minutes. Individual fishes were placed onto a tray together with a PIT tag reader on which the PIT tag ID was displayed with a ruler and photographed with a camera (Nikon DSLR D5100). The BW, standard length and PIT tag ID and additional morphometric data of each fish was recorded from the resulting photograph (for two representative pictures, see **S1 Fig**). This procedure was subsequently repeated twice: at 139 dph when the density was reduced to 50/canvas by removing 25 individuals randomly from each canvas and at the end of the experiment at 168 dph. Length measurements of every individual fish were made subsequently from the images using ImageJ software [37]. Growth rate of fishes was calculated as specific growth rate (SGR) with the following equation: **SGR (% day** ^**-1**^ **) = (lnWf—lnWi)/t × 100**, where Wf is final body weight, Wi is initial body weight and t is the period of growth in days. Feed Conversion Efficiency (FCE) was calculated as (**increase in wet biomass) / (total amount of feed fed) × 100**. Fulton’s condition factor was calculated with the simplified formula based on standard body length (SL) and body weight (BW): **BW/SL** ^**3**^ * **100** [38].

At 168 dph, samples from 18 randomly selected individuals were collected from each group (**Fig 1**). The samples used for molecular analysis and histology were collected from posterior part of the liver and mid-section of gently squeezed intestine, respectively. Muscles from the vicinity of the dorsal fin and tail fin were collected for biochemical analysis. Samples collected for histology were stored in 10% (v/v) buffered formaldehyde at 4°C for at least 48 hours. All collected samples, except the histological ones, were snap-frozen in liquid nitrogen and stored at -80°C prior to use.

### 2.6 Histology and microscopy

Fixed mid-gut and posterior liver tissues were dehydrated in an ethanol gradient (50%, 60%, 70%, 85%, 90% and 96%). Dehydrated tissues were embedded in hydroxyethyl methacrylate (Historesin^®^, Leica, Heidelberg, Germany) and sectioned into a series of 30 sections per sample (section thickness ~4 μm). Sections were mounted on slides and stained using haematoxylin-eosin (H/E). To ensure that H/E could be used for counting of goblet cells, Periodic Acid-Schiff and Alcian blue staining were performed on other sections of the same sample for verification (**S2 Fig**).

Microscopic analyses were performed using a Zeiss Axioplan 2 microscope mounted with a Nikon digital camera DXM 1200F. For mid-gut and posterior liver analysis, nine samples were collected from each group (total of 36 samples). For every gut sample, ten cross-sections were quantified for external circumference of serosa (ECS), mucosal height (MH) and muscularis layer thickness (MLT). Hepatocyte diameter (HD) was quantified by taking thirty measurements per liver section. All measurements were performed using the Fiji software [39]. The goblet cell number (GCN) was determined by counting them in the entire mucosal region under the microscope. Each sample was assessed twice by two researchers and averaged before using it as a representative measurement.

### 2.7 Analysis of the chemical composition of fish fillets

The analyses of dry matter, crude fat and crude protein in fillets were outsourced to ALS Technichem (S) Pte. Ltd. (Singapore). A total of five pools containing 250g of fillets of each group were analysed. FAME synthesis was performed in-house with the method described by O’Fallon and colleagues [40], with minor modifications in the sample procedure. Two and a half gram of the muscle tissues collected from the area on the left side below the dorsal fin were freeze-dried and homogenized by grinding with mortar and pestle under liquid nitrogen. Powdered tissue samples were placed into a 21 X 70 mm glass vial with PTFE screw cap and the subsequent steps of FAME synthesis were performed following the described protocol. After FAME synthesis, the clear hexane layer containing the FAME was placed into a gas chromatography (GC) vial without disturbing the interphase and stored at -20°C prior to GC analysis. FAME was detected by capillary GC on a HP-88 capillary column (Agilent, p/n 112-88A7) installed in an Agilent 6890N network gas chromatograph (Agilent, Santa Clara, CA, USA) that is equipped with a flame ionization detector and split injection. The initial oven temperature of 140°C was gradually increased to 240°C at a rate of 4°C/min. Helium was used as the carrier gas. Fatty acids were identified by comparing their retention times with Supelco^TM^ 37 component FAME Mix standards (47885-U) and individual fatty acids are represented by area % of total fatty acids. The correlation of fatty acid between fillets of groups (Group A-D) and their respective feed type (Feed A-D) were analysed with Pearson’s correlation coefficient. Only fatty acid profiles of big size commercial feeds were used for this correlation. Since FAME of feeds and that of the fillets were detected independently, only fatty acids that were detected in both instances (**S2 Table**) were used for the correlation studies in (**S3 Table**).

### 2.8 Microarray hybridization and analysis

Total RNAs were extracted from liver tissues (eight individuals per feed type) using RNeasy Mini kit with DNAse treatment (Cat No. 74106; Qiagen Singapore Pte. Ltd., Singapore) following the manufacturer’s protocol. RNA concentration was determined using NanoDrop 1000 (NanoDrop Technologies). The RNA integrity number (RIN) of extracted total RNA was determined by using Agilent 2100 Bioanalyser (Agilent Technologies, Nærum, Denmark). Only samples with RIN >8.5 were used for the analysis. Microarray-based transcriptomics were carried out using Agilent SurePrint G3 custom gene expression microarrays (8X60K; Cat No. G4102A) with probes covering an estimated ~70% of the seabass transcriptome (estimated based on BLASTX-search against the Nile tilapia RefSeq protein dataset). The microarray design (**GPL17855**) and probe set analysis data have been deposited into the NCBI Gene Expression Omnibus database (**GSE55152**). Partek® Genomic Suite (v6.6) was used to analyse both sets of data. Microarray data were quantile-normalized and log_2_-transformed for statistical analysis. A three way-ANOVA analysis employing step-up false discovery rate (FDR) multiple test correction (P_fdr_ < 0.05; arbitrary fold change cut-off of 2) was used to identify differentially expressed transcripts / genes (DETs / DEGs). Significantly expressed transcripts (SETs) were identified by unpaired, two-tail T-Test (P < 0.05). Gene Ontology (GO) annotation was performed through AgBase-Goanna [41], GO enrichments were performed in agriGO’s Singular enrichment analysis [42] and visualisation were performed with REVIGO [43]. KEGG Automatic Annotation Server (KAAS) bi-directional best hit method were performed for genes of interest [44] and enrichment analysis of KEGG metabolic pathways was done with GSEA of broad institute [45]

### 2.9 Validation of liver microarray results using Fluidigm qPCR

The expression level of 13 selected DEGs identified earlier by microarray analysis of the liver was quantified by qPCR. In addition, the expression level of Δ6 fatty acyl desaturase (*fads2;* for a detailed list of genes validated, see **S4 Table**) and elongation of very long fatty acid 5 (*elovl5a*) that are typically responsive to dietary n3-lcPUFA were also determined. Primers (~20mers) were designed using NCBI/Primer-Blast (http://www.ncbi.nlm.nih.gov/tools/primer-blast/) and synthesized by Sigma-Aldrich. Five reference genes (*tuba*, *ef1α*, *gadph*, *rpl8* and *ubq*) that have shown stable expression levels over a variety of tissues in Asian seabass were selected as candidate reference genes. Four biological samples from each of four groups (A-D) were reverse transcribed into cDNA using the iScript^TM^ Reverse Transcription Supermix (Cat No. 170–8841; Bio-Rad Laboratories) according to the manufacturer’s instructions. qPCR was performed on a 48.48 Dynamic Array Integrated Fluidic Circuit (IFC) according to the EvaGreen DNA Binding Dye protocols (Fluidigm, CA, USA) resulting in 2,304 simultaneous qPCR reactions, respectively. Expression data were acquired using the Real-Time PCR Analysis software 3.0.2 (Fluidigm). Data pre-processing, normalization, relative quantification, and statistics were performed using GenEx5 (MultiD, Göteborg, Sweden). PCR efficiency were determined by Real-time PCR miner [46]. The most stably expressed reference genes (*ubq* and *rpl8*) were identified from the panel of putative reference genes using GeNorm [47] and their geometric means were used to normalize all samples in GenEx5. Data were log_2_ transformed to attain a normal distribution prior analysis of variance (ANOVA).

### 2.10 Statistical analysis

Data of production traits, histological and biochemical analysis are represented as means values with standard error of the mean (SEM). Production traits of every individual fish were taken into account with the exception of those fishes, which had died (20 individuals) or were problematic (28 individuals) at some point throughout the course of the experiment. Fishes were classified as problematic when unforeseen circumstances occurred such as, missing tag number, missing weight and length measurements. Potential outliers were verified by procedure of Tukey [48] by categorizing ‘outside’ values and confirmed outliers were removed from subsequent analysis. All data were analysed from samples at 168-dph by ANOVA. Post-ANOVA multiple comparisons between the mean of each group with mean of every other group were performed by Tukey’s honestly significant difference (HSD). Post-ANOVA multiple comparisons between the mean of each group with the mean of the control group were performed by Dunnett’s test. Differences were considered significant only, if their adjusted P-value (P_adj_) was < 0.05 with 95% confidence interval. The different strengths of Pearson correlation coefficient (r) were defined as very high, high, moderate, low and negligible correlation [49].

All statistical analyses were performed and graphs were generated using GraphPad Prism version 6.02 for Windows (GraphPad Software, La Jolla, CA, USA; www.graphpad.com) and Microsoft Excel 2010 (Microsoft, Redmond, WA, USA).

## Results

### 3.1 Asian seabass fed pelleted feeds grew faster than controls, but at different rates

Biochemical analysis of the feeds (**Table 1**) re-confirmed most of the wide-ranging differences observed among the composition of the feeds that were expected from the nutrition labels provided on the packaging (**S1 Table**). However, some discrepancies were found for Feed Bb (Feed B, big size) and both Feeds Cs and Cb (Feed C, small and big size). For Feed Bb, the percentage of crude fibre was found nearly half of what was provided on the label, whereas the result of analysis for Feed C showed a 59% and 12% increase in the percentage of crude fat and crude ash, respectively (**S1 Table**).

**Fig 2:**
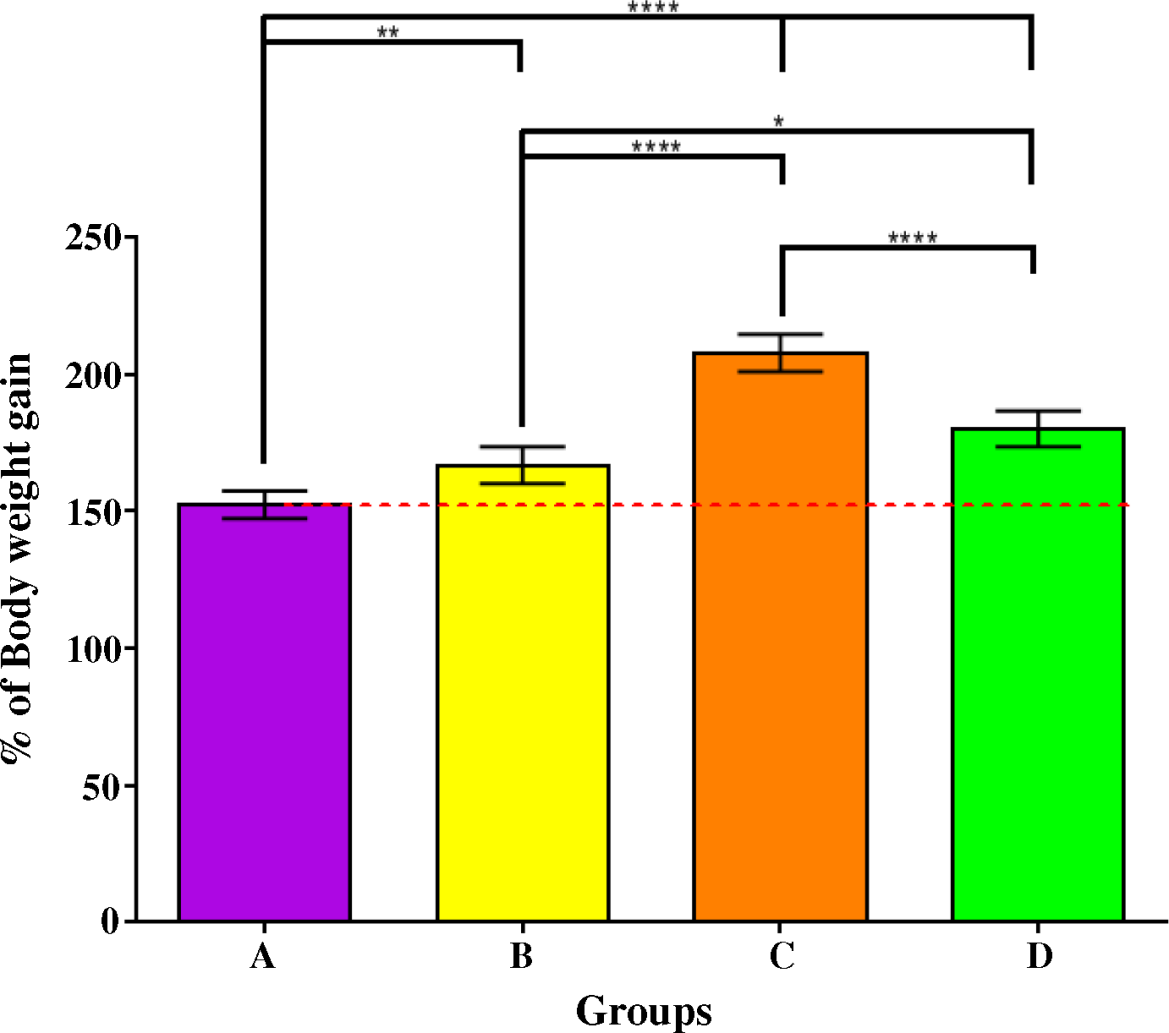
Wide-ranging differences among the relative body weight gains in Asian seabass fed pelleted feeds compared to control. Total percentage of body weight gain after the consumption of three different pelleted feeds (Feeds B-D) relative to frozen baitfish (Feed A) during a two-month period (107–168 days post-hatch). Post-ANOVA Tukey's multiple comparison test was performed to compare the mean values of every group. Error bars are plotted with 95% confidence intervals from the mean. Significantly different means are noted with *—P_adj_ < 0.05; **—P_adj_ < 0.01; ***—P_adj_ < 0.001; and ****—P_adj_ < 0.0001.

After 61 days of feeding, percentage of body weight gain of the groups fed commercial feeds (B-D) were significantly higher than that of the control (Group A; P_adj_ < 0.01) with Feed C having the largest overall growth (**Fig 2**). Statistically significant differences in specific growth rate (SGR) were observed as well among the different groups (A-D) with Group C having the highest SGR of 1.79. However, for standard body length gain, no such differences could be observed between Groups B and A, or between Groups B and D. Group C grew the longest by 0.11 cm per day (**Table 3**). Similarly, no statistical significant differences could be observed for daily body weight gain, feed conversion efficiency (FCE) and condition factor between Groups B and D. The FCE for control (Group A) was only 32.1% (commercial feeds: 91.2% - 101.6%). This was presumably due to the fact that the dry matter content of frozen fish fed was only 26% (**Table 1**), and therefore, nearly thrice the amount of that had to be fed compared to commercial feeds. No significant differences in the mortality have been observed among the different groups.

**Table 3:** Production traits at 168 dph of juvenile Asian seabass fed different feeds (A-D) for 61 days.

Production Traits	Diet (Mean)				SEM			
	A	B	C	D	A	B	C	D
Initial weight (g)	77.5^a^	79.0^a^	78.8^a^	77.9^a^	1.20	1.20	1.20	1.10
Final weight (g)*	196.2^a^	211.7^b^	240.5^c^	213.7^b^	3.20	3.20	3.70	2.90
Daily weight gain (g)*	1.89^a^	2.10^b^	2.58^c^	2.18^b^	0.04	0.04	0.04	0.03
Initial std length (cm)*	12.5^a^	12.8^b^	12.6^ab^	12.7^ab^	0.07	0.07	0.07	0.06
Final std length (cm)*	18.4^a^	18.5^ab^	19.4^c^	18.7^b^	0.10	0.09	0.10	0.09
Daily length gain (cm)*	0.09^a^	0.09^a^	0.11^b^	0.10^c^	0.00	0.00	0.00	0.00
SGR (%)*	1.47^a^	1.55^b^	1.79^c^	1.63^d^	0.02	0.02	0.02	0.02
FCE (%)*	32.1^a^	92.7^b^	101.6^c^	91.2^b^	0.63	1.79	2.40	1.75
Condition factor (K)*	3.11^a^	3.26^b^	3.27^b^	3.22^b^	0.01	0.02	0.02	0.01
Mortality (%)	2.4^a^	0.8^a^	1.9^a^	0.5^a^	1.80	0.50	0.90	0.30

Results are mean ± SEM. Significant differences between all the different feed types were determine by one way ANOVA indicated by (*, P < 0.05). Post ANOVA Tukey's multiple comparisons test was done to comparing between the mean values for each of the different diets. Means with different letter of superscripts are significantly different, (P_adj_ < 0.05). SEM = Standard Error of Mean; std = standard; SGR = specific growth rate; FCE = feed conversion efficiency. The formulae for SGR, FCR and Condition factor could be found in subsection 2.5 under Materials and Methods.

### 3.2 Fillet-based fatty acid profiles of fishes fed pelleted feeds were distinctly different from those of controls

The proximate nutrient content of juvenile Asian seabass fillets did not vary significantly among the four groups, except for the percentage of crude fat. Consumption of Feed B seemed to result in a statistically significant increase in accumulation of crude fat in the fillet compared to the controls (P_adj_ < 0.05; **Table 4**).

**Table 4:** The proximate nutrient content and fatty acid composition of flesh of Asian seabass juveniles on different diets at 168 dph.

**(A) Proximate nutrient composition**	**Diet (Mean)**				**SEM**			
	**A**	**B**	**C**	**D**	**A**	**B**	**C**	**D**
Dry matter (g/100 g)	21.7^a^	23.0^a^	23.5^a^	23.1^a^	0.68	1.0	1.6	1.1
Crude protein %	21.0^a^	20.6^a^	18.9^a^	20.4^a^	0.19	0.68	0.87	0.85
Crude fat %	1.0^a^	2.3^b^	1.6^ab^	1.4^ab^	0.07	0.25	0.14	0.07
**(B) Fatty acid composition (area %)**	**Diet (Mean)**				**SEM**			
	**A**	**B**	**C**	**D**	**A**	**B**	**C**	**D**
Myristic (C14:0)	2.5^a^	3.9^b^	2.0^a^	3.6^b^	0.13	0.12	0.06	0.19
Pentadecanoic (C15:0)	0.3^a^	0.3^a^	0.2^b^	0.3^a^	0.03	0.01	0.02	0.03
Palmitic (C16:0)	18.9^a^	18.8^ab^	18.2^b^	21.0^c^	0.22	0.22	0.10	0.16
Heptadecanoic (C17:0)	0.8^a^	0.4^b^	0.3^b^	0.5^ab^	0.15	0.06	0.05	0.07
Stearic (C18:0)	8.7^a^	6.4^b^	6.8^bc^	7.3^c^	0.19	0.16	0.15	0.20
Arachidinic (C20:0)	0.2^a^	0.2^a^	0.2^a^	0.2^a^	0.02	0.01	0.02	0.02
ΣSFA	31.5^a^	30.1^b^	27.7^c^	33.0^d^	0.32	0.28	0.14	0.25
Palmitoleic (C16:1)	3.2^a^	5.0^b^	3.2^a^	4.7^b^	0.16	0.13	0.07	0.20
cis-10 Heptadecanoic (C17:1)	0.7^a^	0.9^b^	0.6^c^	0.8^d^	0.01	0.02	0.01	0.01
Oleic (C18:1n9c)	16.1^a^	19.3^b^	28.9^c^	17.8^d^	0.41	0.29	0.36	0.16
cis-11 Eicosenoic (C20:1)	1.0^a^	0.8^ac^	1.3^b^	0.7^c^	0.06	0.04	0.02	0.02
nervonic acid (C24:1n9)	0.3^a^	0.2^a^	0.2^a^	0.2^a^	0.07	0.05	0.04	0.02
ΣMUFA	21.3^a^	26.3^b^	34.2^c^	24.2^d^	0.54	0.36	0.42	0.24
Linoleic (C18:2n6)	4.9^a^	10.5^b^	14.5^c^	8.2^d^	0.21	0.21	0.21	0.10
γ-Linolenic (C18:3n6)	0.2^a^	0.4^b^	0.4^b^	0.3^b^	0.02	0.01	0.00	0.03
cis-11,14 Eicosadienoic (C20:2 n6)	0.2^a^	0.2^a^	0.2^a^	0.2^a^	0.02	0.02	0.02	0.02
cis-8,11,14 Eicosatrienoic (C20:3 n6)	0.2^a^	0.2^a^	0.2^a^	0.2^a^	0.02	0.02	0.02	0.03
Arachidonic (C20:4n6)	3.6^a^	2.0^b^	1.6^c^	2.6^d^	0.15	0.07	0.05	0.09
Σn6-PUFA	9.2^a^	13.2^b^	17.0^c^	11.6^d^	0.17	0.16	0.17	0.08
α-Linolenic (C18:3n3)	1.0^a^	1.4^b^	1.9^c^	1.2^d^	0.06	0.05	0.05	0.03
cis-11,14,17 Eicosatrienoic (C20:3 n3)	0.5^a^	0.4^a^	0.4^a^	0.4^a^	0.06	0.03	0.02	0.03
cis-5,8,11,14,17 Eicosapentaenoic (C20:5n3)	4.6^a^	8.8^b^	3.2^c^	6.4^d^	0.06	0.16	0.09	0.05
cis-7,10,13,16,19 Docosapentaenoic (C22:5n3)	3.0^a^	2.9^a^	1.5^b^	2.5^c^	0.08	0.07	0.03	0.03
cis-4,7,10,13,16,19 Docosahexaenoic (C22:6n3)	21.1^a^	9.8^b^	8.8^b^	13.2^c^	0.44	0.31	0.35	0.30
Σn3-PUFA	30.2^a^	23.4^b^	15.9^c^	23.8^b^	0.36	0.27	0.41	0.33
ΣPUFA	39.3^a^	36.6^b^	32.8^c^	35.3^d^	0.32	0.19	0.28	0.33
Σn6/Σn3	0.30^a^	0.57^b^	1.08^c^	0.5^b^	0.01	0.01	0.04	0.01

(A) Mean values for proximate nutrient composition are represented based on wet matter basis.

(B) Mean values for fatty acids composition are represented in area % of total composition or fatty acids. Means with different letter of superscripts are significantly different, (P_adj_ < 0.05).

SFA = Saturated fatty acids; MUFA = Monounsaturated fatty acid; PUFA = Poly unsaturated fatty acid.

**Fig 3:**
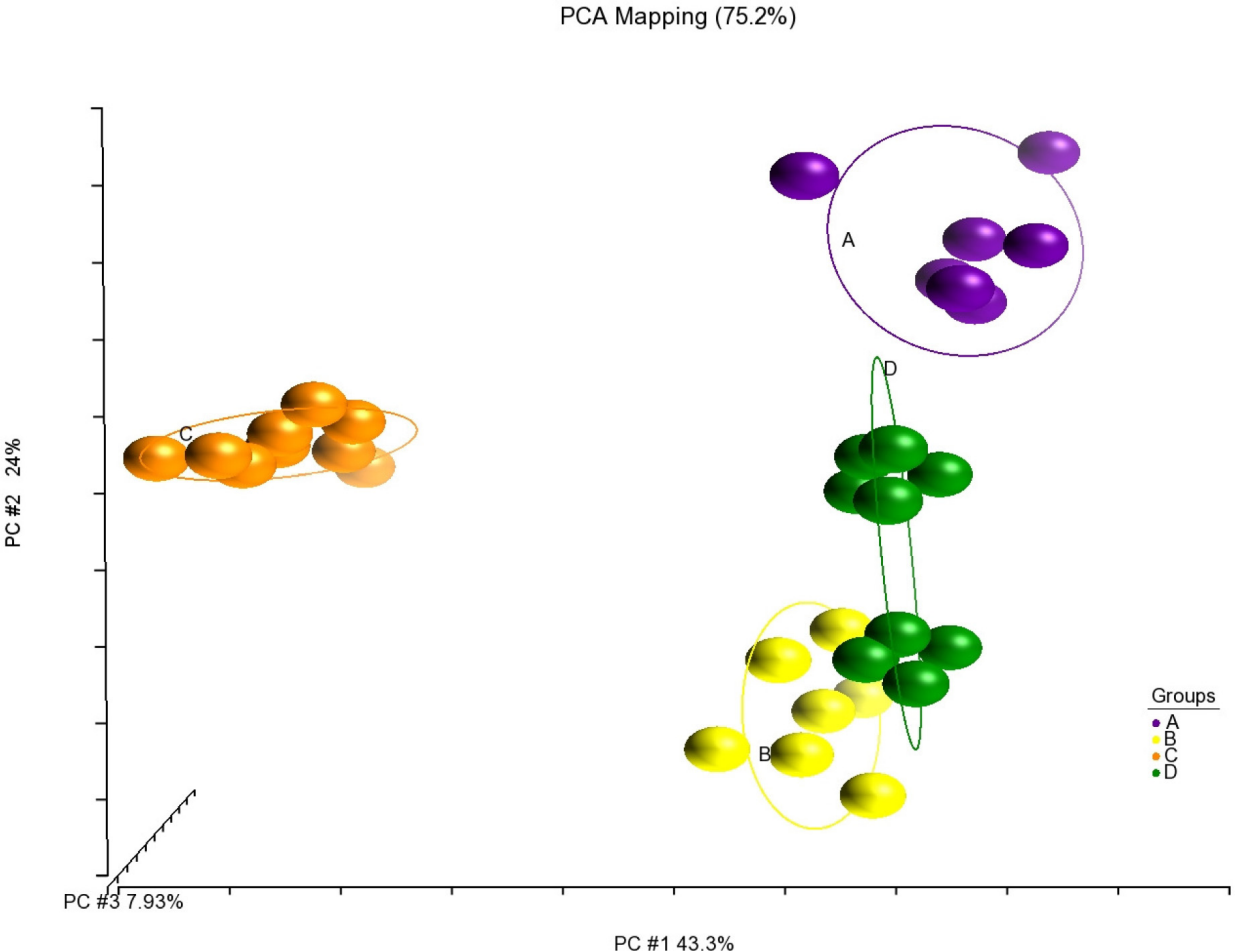
Formation of unique clusters of fillet fatty acid profile after consuming different pelleted feeds (Feed B-D) and frozen baitfish (Feed A). The PCA plot generated by Partek® Genomic Suite (v6.6) is based on covariance matrix of 21 fatty acids (area % of total fatty acids) per sample. Coloured balls represent individual samples fed with different feed types.

Fatty acid analysis of juvenile Asian seabass fillets showed that the consumption of different feeds had altered the Saturated Fatty Acid (SFA), Monounsaturated Fatty Acid (MUFA) and PUFA composition in a uniform manner. Fillets originating from groups fed with different feed types formed clear distinctive clusters on the PCA plot (**Fig 3**). The fatty acid profiles of Group B and C were different from that of Group A, whereas Group D was more similar.

However, despite having their unique clusters, the predominant fatty acids of the fillets from groups fed different commercial diets were similar. Palmitic acid (C16:0), oleic acid (C18:1n9c), linoleic acid (LA, C18:2n6c) and DHA (C22:6n3) were predominant for SFA, MUFA, n6-PUFA and n3-PUFA, respectively (**Table 4**). The fillets from Group C had the highest ratio of n6/n3 at 1.08 with their fatty acid composition containing the most LA and least DHA content when compared to other groups.

### 3.3 Fatty acid composition of the fish fillets mirrored that of the corresponding feeds

**Fig 4:**
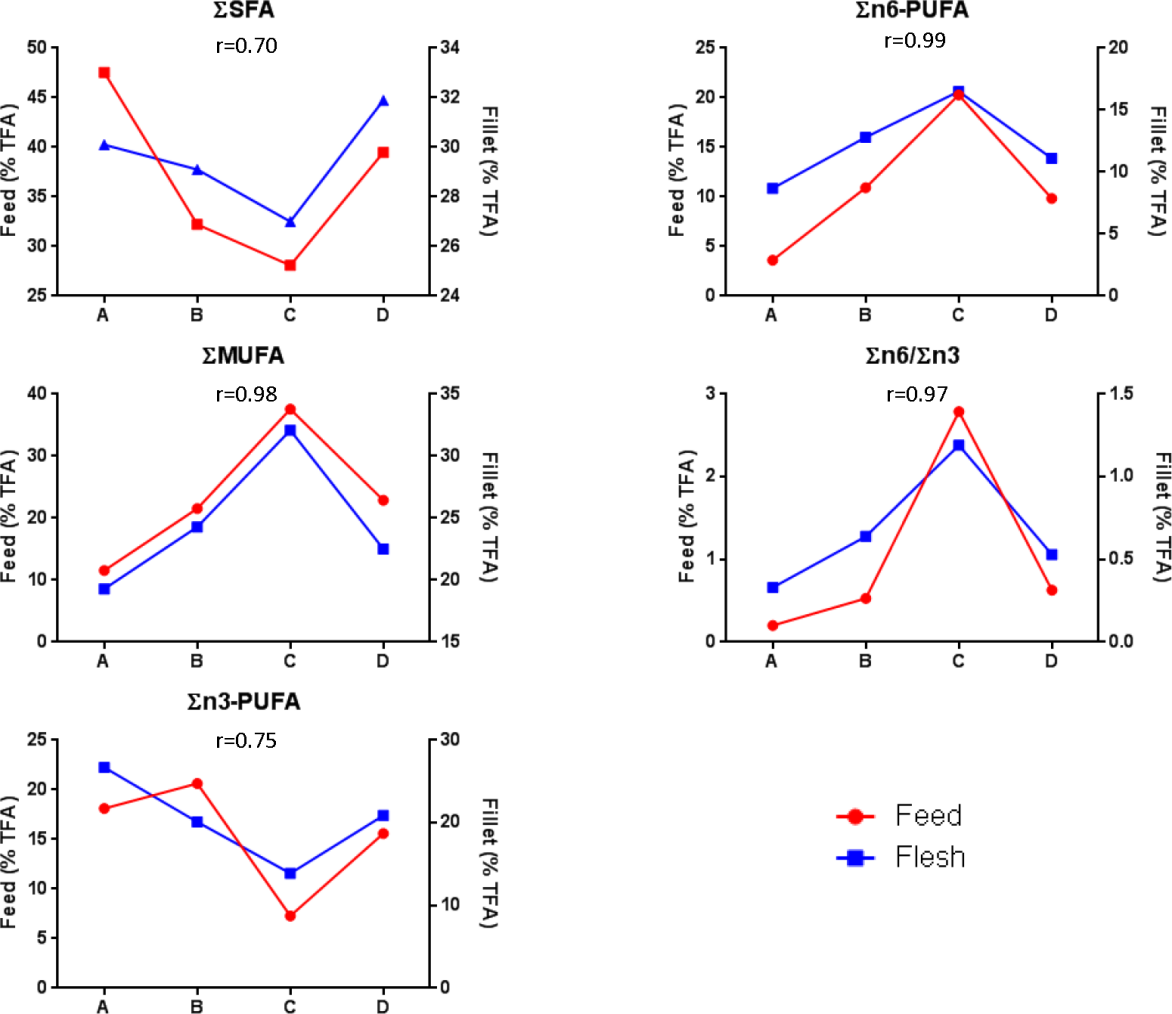
Positive correlation of fatty acids between fillets of groups and their respective feeds. Only area % of TFA of large-sized pelleted feeds were used for correlation. Pearson’s correlation coefficient (r) was used to determine the correlation relationship. TFA = Total Fatty Acids.

When fatty acid compositions that were detected in the fillets of the different groups (A-D) and their respective feed types (**S2 Table**) were compared, the graphs displaying the different proportion of fatty acids types closely mirrored each other showing a high positive correlation (r ≥ 0.7; **Fig 4**). The highest and lowest correlation coefficient was observed in ΣMUFA (r = 0.98) and ΣSFA (r = 0.70), respectively (**Fig 4**).

Interestingly, when comparing the combined data of all fillets and that of all feeds, every individual fatty acid correlated positively from a moderate correlation (C14:0; r = 0.06) to very high correlation (r = 1.0) with the exception of γ-linolenic acid (GLA, C18:3n6) where negligible correlation was found (r = 0.1) (**S3 Table**). Further analysis of the individual fatty acids revealed two distinct relationships of clusters among fillet and feed. Cluster 1 consisted of arachidonic acid (ARA, C20:4n6), EPA, DHA, C16:1, C14:0, C16:0 and C18:0, whereas Cluster 2 consisted of α-linolenic acid (ALA, C18:3n3), LA and oleic acids (OA, C18:1n9c). It was observed that the Cluster 1 and Cluster 2 fatty acids of feed and fillet correlated either positively or negligibly within their own cluster, but between the two clusters only either negative or negligible correlation were observed. The only exception was GLA (C18:3n6) that did not fit into either of the two clusters (**S3 Table**).

### 3.4 Distinct histological changes were observed in the mid-gut and liver of fish fed pelleted feeds compared to controls

**Fig 5:**
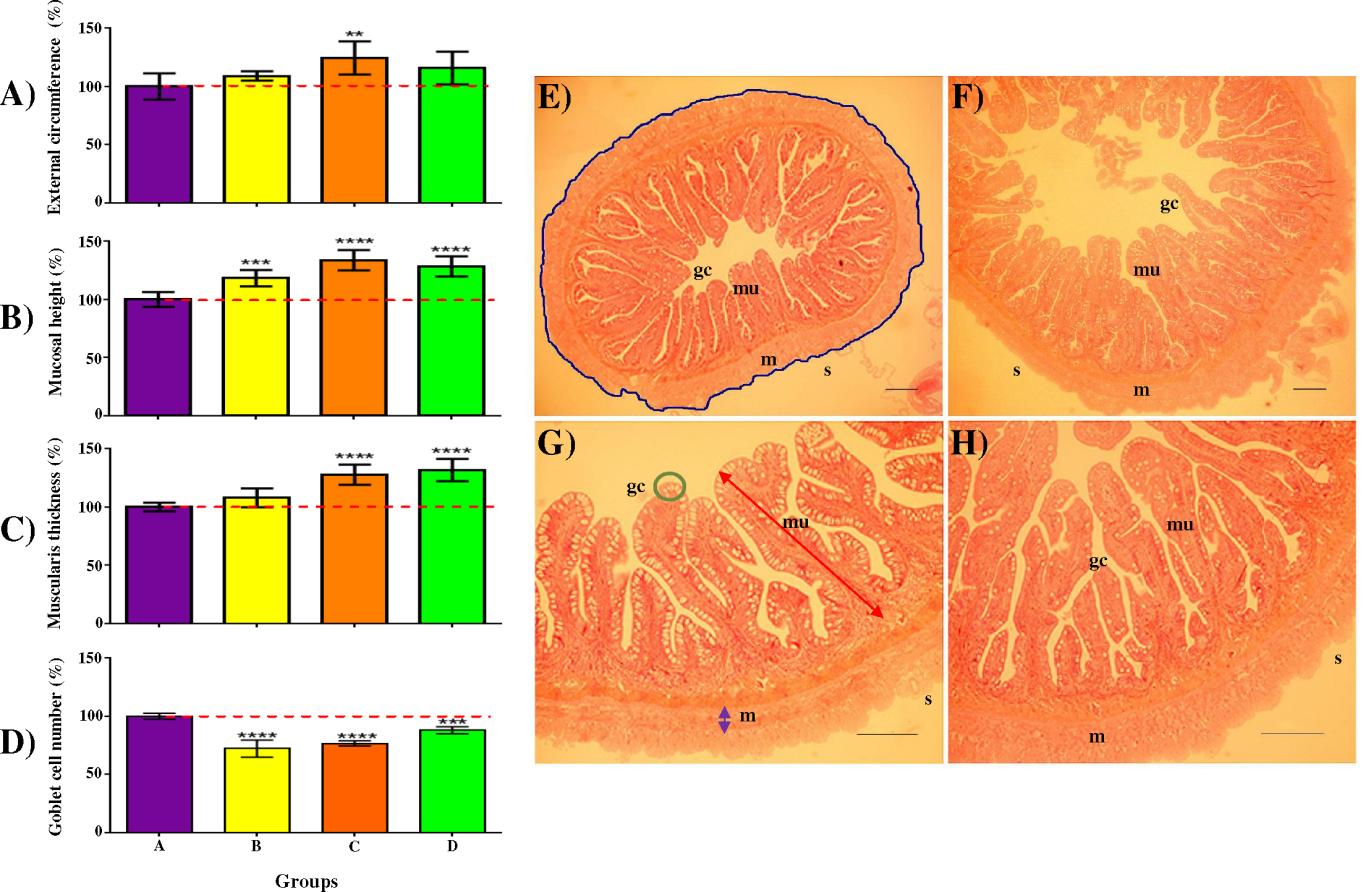
The gastrointestinal tract of Asian seabass is influenced by the different feeds. External circumference of serosa (Panel A), mucosal height (Panel B), muscularis layer thickness (Panel C) and goblet cell number (Panel D) were measured from sections of the mid gut of 168 dph-old Asian seabass fed frozen baitfish (Group A) or pelleted commercial feeds (Groups B-D) for 61 days. Values are represented as percentages over control (A) value. Post ANOVA Dunnett’s multiple comparisons test was performed between the mean of each group with the mean of the control group. Means significantly different from Group A are noted with *—P_adj_ < 0.05; **—P_adj_ < 0.01; ***—P_adj_ < 0.001; or ****—P_adj_ < 0.0001. Representative examples of the mid-gut histological sections are shown on the right (Panels E&G–Group A; F&H–Group C). Panel E: Blue line = External circumference of serosa (S); Panel G: red = high of Mucosa (mu), purple = Thickness of muscularis layer (m), green = Goblet cells (gc); Panels E&F - 5x magnification; Panels G&H - 10x magnification. Panels E&H; scale bar = 100μm.

Histological parameters were examined in order to detect potential morphological modifications in the intestine due to different dietary intakes. From the mid-gut cross section of commercial feed fed groups (B-D), significantly longer ECS values were observed in Group C (~9.4 mm)–but not for other groups when compared to Group A (~7.5 mm; **Fig 5A and S5 Table**). Groups C and D had significantly thicker MLT and all three commercially fed groups (B-D) showed significant increase in MH when compared to Group A (**Fig 5**). The profiles for GCN showed a reversed trend, Asian seabass fed pelleted feeds had significantly lower number of goblet cells than controls (Group A) fed frozen fish (**Fig 5D and S5 Table**). The possibilities of the different histological parameters correlating with body weight (BW) were cross-checked for all 36 samples and negligible correlation was observed for GCN, low positive correlation was observed for ECS and MH, while moderate positive correlation was observed for MH (**S3 Fig**).

**Fig 6:**
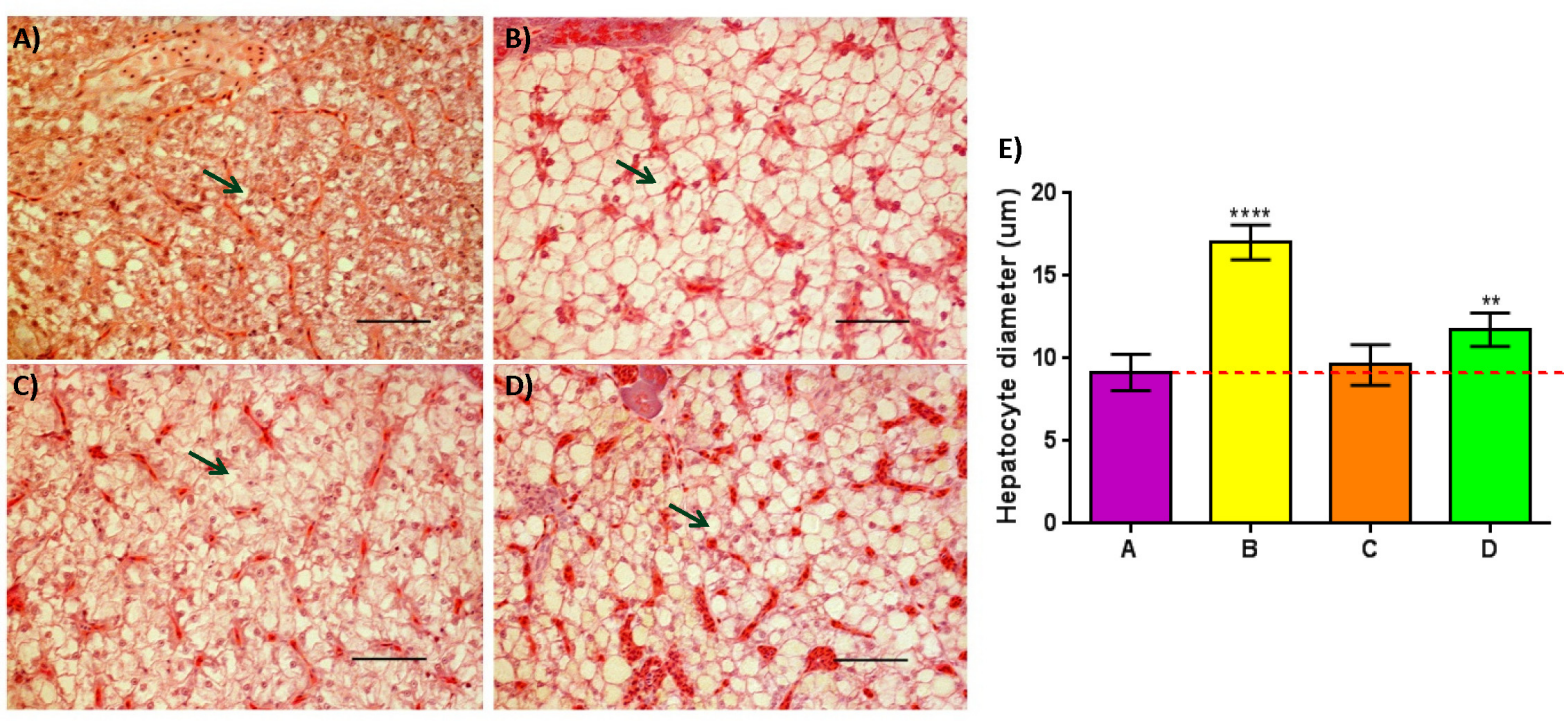
Increased lipid deposition observed in two of the three pellet-fed groups compared to controls with the exception of Group C. Panel (A-D): cross sections of posterior liver stained with H/E of Groups (A-D) respectively; Green arrows = hepatocytes containing lipid droplet. Panel (a-d); 40x magnification, scale bar = 50μm. Panel E: Quantitative analysis of mean hepatocyte diameter detected within Groups (A-D); Post ANOVA Dunnett’s multiple comparisons test was performed between the mean of each group with the mean of the control group. Means significantly different from Group A are noted with (*—P_adj_ < 0.05; **—P_adj_ < 0.01; ***—P_adj_ < 0.001; ****—P_adj_ < 0.0001).

Visually, hepatocytes of all the groups fed commercial feeds appeared to be enlarged when compared to Group A (**Fig 6A–6D**). However, when the average diameter of the hepatocytes was determined, a significant enlargement of hepatocytes within the posterior part of the liver compared to control was only observed in Groups B and D (P_adj_ <0.01), but not in Group C (**Fig 6E**).

### 3.5 Transcriptomic analysis revealed differential expression of hepatic genes and metabolic pathways in the liver of pellet-fed fish compared to controls

Microarray-based transcriptomic analysis was performed to assess the potential effects of the four different feeds (Groups A-D) in the hepatic samples. The overall expression profile of each of the pellet-fed groups (B, C and D) showed a small, variable number (7 to 127) of differentially expressed transcripts (DETs; P_fdr_ < 0.05, 2 fold-change limit) when compared against the baitfish-fed group (A). On the other hand, DETs between the pellet-fed groups could only be detected when a less stringent statistical method was applied with higher Type 1 error rate (multiple T-test). Under such circumstances, a few thousand significantly expressed transcripts (SETs; P < 0.05, no fold change limit) were observed (**S4 Fig**).

From the ANOVA analysis, 39 transcripts were found to have significantly different expression level (P_fdr_ < 0.05; no fold change limit) among the different groups (A-D). These transcripts, when plotted on a heat map (**S5A Fig**), summarised the overall hepatic transcriptomic patterns. Group C had a very similar profile to that of baitfish-fed control (Group A). Specific transcripts were observed to be significantly up-regulated in pellet-fed groups (B, C and D), but down-regulated in control and this was illustrated when the subset of the different DETs were plotted (**S5B Fig**). Four particularly interesting transcripts that encoded for: *fads6*, LOC102313855, LOC102081928 and a novel unknown transcript, respectively, were found to be able to distinguish pellet-fed groups (B, C and D) from controls (A).

**Fig 7:**
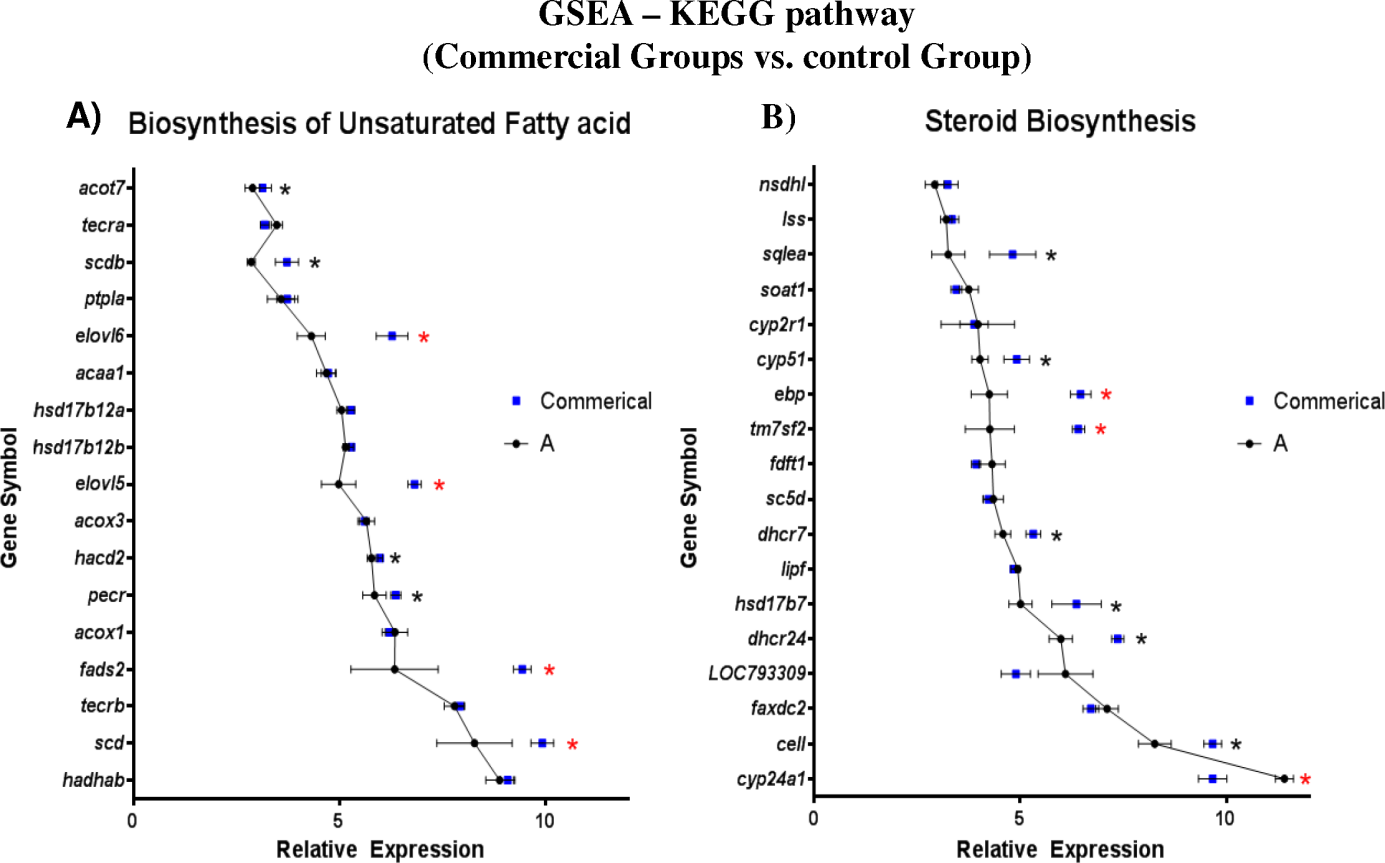
Gene Set Enrichment Analysis (GSEA) of KEGG pathway comparing pellet-fed groups (B, C and D) against baitfish-fed control (Group A). By using *Danio rerio* as reference model, (A) genes that are involved in Biosynthesis of Unsaturated fatty acid pathway, (B) genes that are involved in steroid biosynthesis pathway were compared. Significantly enriched genes are noted with *—P < 0.05. Out of those *, genes that are differentially expressed as well are marked with *—P_fdr_ < 0.05. Error bars are mean ± SEM.

By performing gene set enrichment analysis on KEGG metabolic pathways using *Danio rerio* as reference model, a different set of pathways were found significantly enriched when pairwise comparison of pellet-fed groups (B, C and D) was performed against the control (A). The most prominently enriched subcategory was ‘Lipid metabolism’ in which, ‘Steroid metabolism’ and ‘Biosynthesis of unsaturated fatty acids’ were found to be consistently enriched in all three comparisons (**S6 Table**). At the level of genes, *elongation of very long fatty acid 5 and 6* (*elovl5* and *elovl6)*, *fatty acid desaturase 2* (*fads2*) and *stearoyl-CoA desaturase* (*scd*) were differentially expressed in the ‘Biosynthesis of unsaturated fatty acids’ pathway, whereas *emopamil binding protein* (*ebp*), *transmembrane 7 superfamily member 2* (*tm7sf2*) and *cytochrome P450*, *family 24*, *subfamily A*, *polypeptide 1* (*cyp24a1*) were differentially expressed in the ‘Steroid metabolism’ pathway (**Fig 7**).

**Fig 8:**
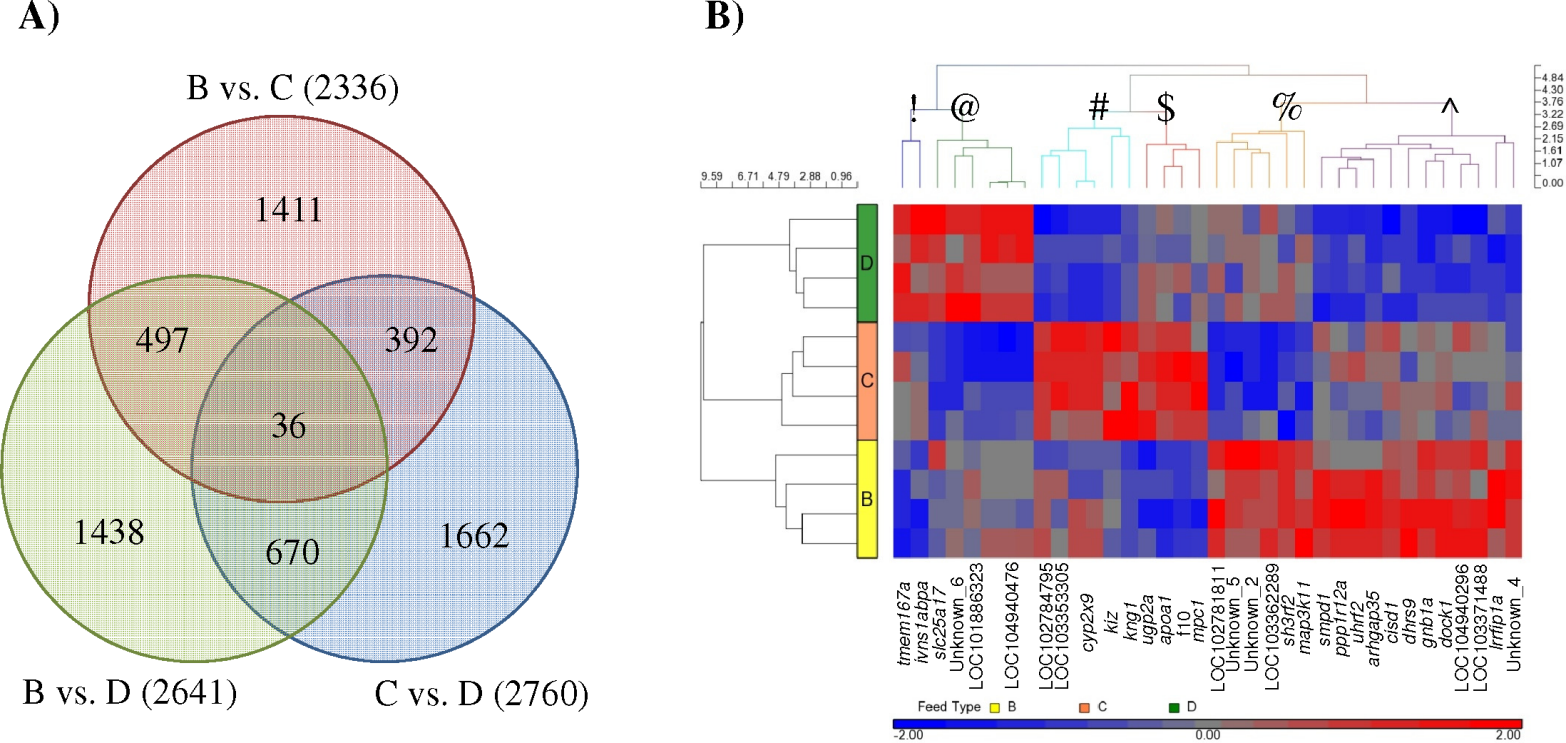
Different sets of significantly expressed transcripts (SETs) with limited overlaps were found when the liver-derived transcriptome of pellet-fed groups (B-D) were compared. (A) Venn diagram showing 36 SETs uniformly different in all three commercial vs. commercial feed comparisons. (B) Heatmap showing the complexity of hepatic expression caused by having different dietary intake. Different clusters of genes are represented by symbols (!, @, #, $, %, ^).

Through multiple T-test analyses, 36 SETs were found commonly expressed between the pellet-fed groups (i.e. B *vs*. C; C *vs*. D and B *vs*. D; **Fig 8A**). The heat map generated from the corresponding 34 genes clearly illustrated the effect of consuming different feeds as six distinct clusters of genes were observed. In cluster #, genes were found to be distinctively up-regulated in Group C, down-regulated in Group D, but displayed mixed expression among individual samples from Group B. Genes in Cluster $ were similarly up-regulated in Group C, but down-regulated in Group B and showed mixed expression in Group D (**Fig 8B**).

**Fig 9:**
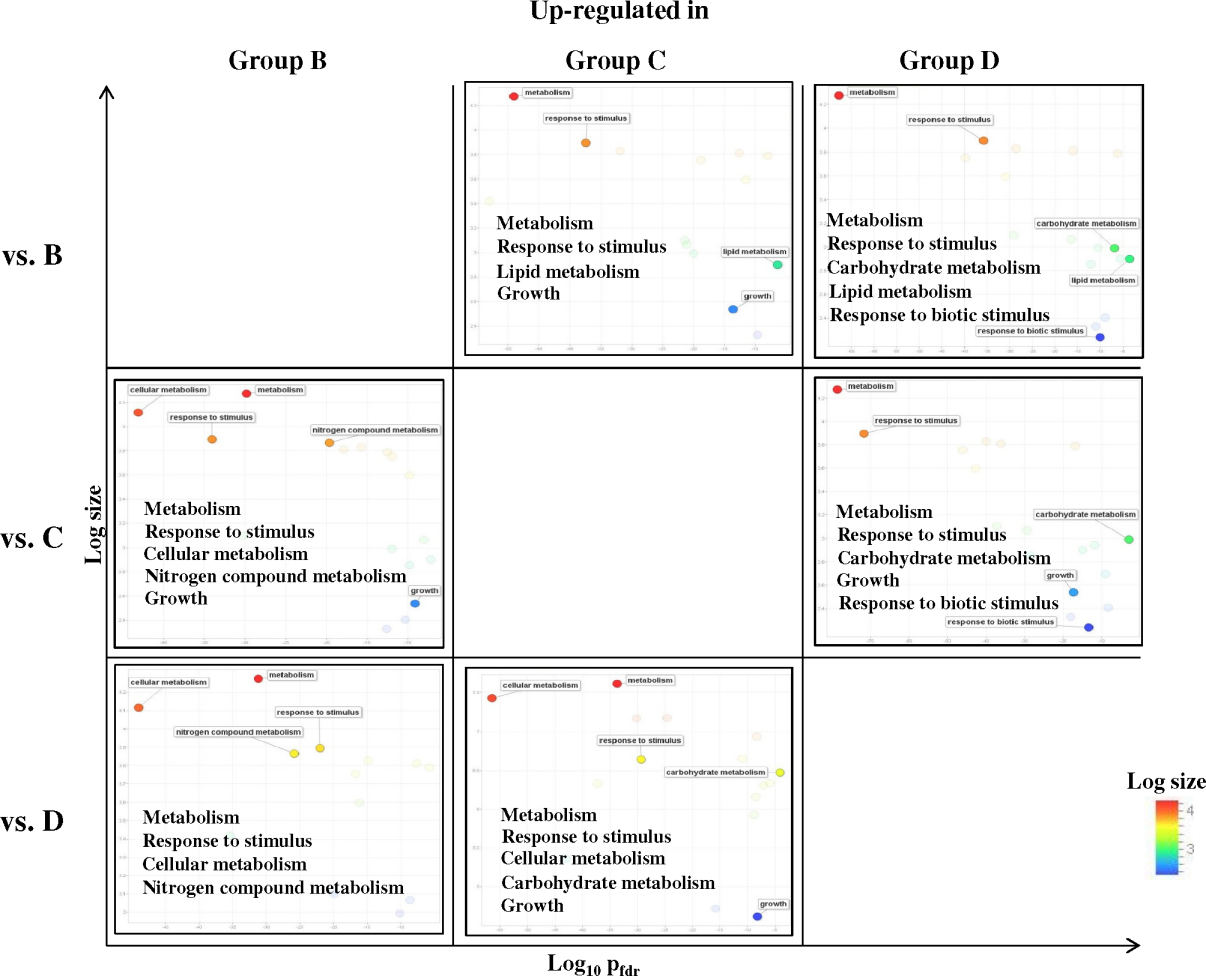
Gene ontology enrichment of significantly up-regulated transcripts between the commercial groups (B-D). Significantly up-regulated transcripts of were analyzed by singular enrichment analysis (agriGO). Results of individual comparisons were presented in a three by three plot. Out of the many ‘Biological Processes’ found to be significantly enriched, those related to metabolism and growth were highlighted and compared upon.

Gene ontology (GO) enrichment for the unique SETs found between the commercial groups (B vs. C: 1,411 transcripts, B vs. D: 1,438 transcripts and C vs. D: 1,662 transcripts; **Fig 8A**) was performed and an interesting phenomenon was observed. By focusing only on significantly enriched biological processes GO (P_fdr_ < 0.05) of up-regulated and down-regulated SETs separately, ‘Metabolism’ and ‘Response to stimulus’ were uniformly found to be enriched in the all comparisons (**Fig 9**). Transcripts involved in ‘Growth’ were enriched only when either Group B or Group D were compared with Group C, but not in the Group B *vs*. Group D comparison. ‘Lipid metabolism’ enrichment was exclusively found up-regulated in Groups C and D when compared with Group B. Up-regulated enrichment of ‘Carbohydrate metabolism’ and ‘Response to biotic stimulus’ was found in Group D when compared to Group B and Group C, but enrichment of down-regulated ‘Carbohydrate metabolism’ transcripts was also detected when Group D were compared with Group C. On the other hand, up-regulated enrichment of ‘Cellular metabolism’ and ‘Nitrogen compound metabolism’ was detected in Group B, but enrichment of down-regulated ‘Cellular metabolism’ transcripts was also found when Group B *vs*. Group C (**Fig 9**).

Microarray results were validated by qPCR. Out of the 60 fold-change differences, 50 (83%) displayed similar expression regulation and only 10 of the fold-changes showed an opposite regulation pattern. However, if observed closely, none of those non-coordinated transcripts had expression fold-change larger than (1.1) or smaller than (-1.1) (**S7 Table**).

## Discussion

### 4.1 Trade-off for growth over fillet quality was observed in pellet-fed Asian seabass

We have analysed the growth performance and fillet quality of Asian seabass fed with three different pelleted feeds by comparing against control (Group A) that was fed with frozen baitfish to mimic conditions in the wild. It has been shown for other food fish species that the fatty acids composition of the fillets correlates positively and is influenced by dietary fats [50–52]. However, most (if not all) of the previous publications were based on specific alterations of the fatty profile by introducing or substituting single [50,53] or multiple [51,52] ingredients of the feed. In this study, by comparing different commercially available feeds without any additional modification, we obtained similar findings made earlier by others for Asian seabass [9,53].

Comparative analysis of the fatty acid profile of fillets clearly showed that pelleted feeds increased the n6/n3 fatty acid ratio of the flesh, in comparison to control (Group A; **Table 4**) and the extent of these changes were different among the groups. Juvenile Asian seabass fed Feed C showed the best growth performance with the highest SGR, FCE and K factor values. On the other hand, the fatty acid composition in the fillet of these fish had the lowest percentage of EPA, DHA and the highest percentage of LA and OA. This led us to suggest that a large proportion of vegetable oil (instead of fish oil) could have been used for the production of Feed C. The large amount of LA found in these fillets seems to support earlier findings that partial replacement of fish oil by vegetable oil would not affect the growth and FCR of juvenile Asian seabass [54]. However, the large quantity of LA and reduced quantity of EPA and DHA had increased the n6/n3 ratio of Group C (> 1), compared to the range of 0.5 observed in the other two groups fed pelleted feeds (B and D). (A note of caution: these feeds may contain an unknown proportion of micronutrients, prebiotics and/or additives. Although they are typically absorbed through the intestine, since metabolism is not always clear, these materials–or their metabolites–might exert their effects on the processes described above.)

In the new millennia, the ratio of n6/n3 PUFA in the typical North-American diet is approximately 10:1 [55] and the major negative health implications associated with having a diet high in dietary n6-PUFA are well documented [56]. n6 fatty acids tend to promote the development of atherosclerosis and metabolic syndrome [57,58], whereas n3 fatty acids have the opposite effect [59], plus the latter were also found to show anti-allergic effects in the gut [60]. In order to compensate for the lack of n3-PUFA in their diet, many consumers increased their seafood consumption as it is supposedly rich in these compounds [61–63]. However, the fatty acid profiles of farmed marine/diadromous fishes are under heavy influence of their dietary intake. As demonstrated by this experiment, if fish farmers were to use a feed with characteristics similar to Feed C to rear Asian seabass, they would indeed produce fish with high growth rate and excellent FCE, but the fatty acid profile of the meat would not be nearly as advantageous to human consumers as that of the wild-caught seabass.

The above observations highlight the importance of considering not only the quantity, but also the quality of the seafood products generated by aquaculture. In our opinion, feeds that allow for moderately increased growth rates with lesser decrease in the quality of fatty acid profiles (e.g. Feed D in our study) should be considered over those that promote even higher growth on the expense of decreased fillet quality (e.g. Feed C).

### 4.2 Identification of potential bio-indicators that could be used to differentiate wild-caught Asian seabass from farmed ones

According to our knowledge, our study is the first to investigate the effect different commercially available pelleted diets on hepatic transcriptome of Asian seabass. Among the DETs detected, the expression level of *fads6*, LOC102313855, LOC102081928 and a novel transcript (unknown_3) seem to have the potential to differentiate wild-caught Asian seabass from farmed ones (**S5B Fig**). Interestingly, both LOC102081928, which is orthologous to human *BTN2A1*, and *fads6* are involved with lipid and fatty acid metabolism [64,65]. Moreover, the former is the ortholog to human *VSIG1*, whose reduced expression may play a role in gastric carcinogenesis [66]. However, additional studies are needed to validate and determine the suitable expression threshold before they can be used for such purpose.

Similarly to the above four genes, *fads2*, *elovl5* and five other additional DEGs found in ‘Biosynthesis of unsaturated fatty acid’ and ‘Steroid biosynthesis’ pathways also seemed to be suitable biomarkers. In Asian seabass, the Δ6 fatty acyl desaturase (Δ6Fad) enzyme has a dual Δ6/Δ8 activity that is capable of utilizing both LA (C18:2n6c) or ALA (C18:3n3) fatty acids as substrates with similar efficiency [10]. However, when expressed in yeast, it was found that the transgenic Δ6Fad enzyme had a preference for n3 over n6 fatty acid-type substrates [67]. Despite the contradiction, both studies confirmed that Asian seabass have the capability to biosynthesize C20:4n3 and C20:3n6 fatty acids from their C18-PUFA derivatives. In our studies, significantly higher expression of *fads2* and *elovl5* that are involved in ‘Biosynthesis of unsaturated fatty acid’ pathway were observed when commercial groups (B-D) were compared against control (Group A). Most of the fillets’ fatty acids, with the exception of GLA, correlated positively with their respective feeds. This showed that the majority of fatty acids found in the fillets are most likely accumulated rather than biosynthesized. Taken together, GLA seemed to be actively produced as a product from LA by Δ6Fad or/and used as a substrate by the Elovl5 to form C20:3n6. Whether Asian seabass is capable of biosynthesizing EPA, DHA and ARA from C20:4n3 and C20:3n6 remains to be determined as attempts to clone and describe the fatty acid desaturase 1 (*fads1*) gene encoding the Δ5Fad enzyme from Asian seabass were unsuccessful [8,9]. Our results provide additional confirmation to earlier reports [10,68,69] that ARA, EPA and DHA are fatty acids that are essential for Asian seabass, similarly to other marine teleosts.

### 4.3 The potential effect of different nutrient content and fatty acid composition of feeds could be visualized through transcriptomic analysis

Although there are several feeds available for Asian seabass on the market, there has not been an integrative approach to assess their beneficial or adversary effects on the growth of fish and the nutritional value of their fillet. In this study, the proximate nutrient composition and the fatty acid profile among the commercial feeds (B-D) were found to vary considerably from one another (**Tables 1 and 2**). When the hepatic expressions of different groups (B-D) were analyzed, no DETs could be found between them. However, when focused on the SETs, complex expression patterns were observed where none of the transcripts was exclusively expressed in a particular group (**Fig 8B**). Genes like *apolipoprotein A-I* (*apoa1*) and *coagulation factor X* (*f10*) in cluster ‘$’ were found to have higher expression in Group C, lower expression in Group B and mixed expression in individuals within Group D. Being the major component of high density lipoprotein (HDL), Apoa1 promotes the transport of cholesterol and phospholipids in humans [70,71]. On the other hand, vitamin-K dependent clotting factor X in healthy young humans is known to be correlated with levels of total cholesterol and low density lipoprotein [72]. Therefore, as shown in this study, the changes in cholesterol metabolic activity among the different groups could be elucidated by transcriptomic analysis. Presumably, they were caused by the different dietary intakes, but one needs to remember that in addition to the quantified macronutrients and fatty acids content, additional nutrients such as prebiotics and/or additives might have also contributed to the observed effects. In addition to ‘Metabolism’ related transcripts that were found to be significantly enriched across all the pellet-fed groups (B-D) (**Fig 9**), transcripts involved in ‘Growth’ are particularly interesting. Significant enrichment of ‘Growth’ related transcripts were found only when Group C was compared against either, Group B or D and no enrichment was found between Group B and D. This trend was seemingly reflected upon the growth profile of the pellet-fed groups. The largest growth differences recorded were between Group C against either, Group B or D, whereas between Group B and Group D, least growth differences were observed. Having found these ‘Metabolism’ related transcripts being significantly expressed and enriched among the commercially fed groups; these highlight the potential impact of having inconsistency among feed manufacturers to produce different types of feeds for a single species.

### 4.4 Histological changes observed in the gastrointestinal tract and hepatic tissues indicated potential negative health effects in fish consuming pelleted feeds

A detailed histological analysis performed on the mid-gut had identified several changes caused by different dietary intake in our Asian seabass (**Fig 5**). Preliminary observation led us to suspect that the differences in histological parameters could be linked to the BW of the fish as the profile for ECS, MH and MLT (**Fig 5A–5C**) seems to be similar to the percentage of BW gain at 168 dph (**Fig 2**). However, upon further investigation, MH showed moderately positive correlation, whereas ECS and MLT had only low positive correlation to BW (**S3 Fig**). These findings suggest that the size of the fish did indeed alter the morphology of the intestine, but only to a limited extent and the differences in feed intake could have play a role in the changes observed. Unlike the rest of the histological parameters, GCN showed negligible correlation to BW and significant decrease in mid-gut sections derived from pellet-fed fish *vs*. the controls were observed (**Fig 5D and S6 Table**). Goblet cells are among the most numerous cells of the gut epithelia, they are the main source of mucin secretion that protects the inner surface of the intestine from pathogens and lubricates the gut content to ease its passing [73]. It has been shown in other teleosts that a variety of dietary factors may attribute to the change in GCN [74,75]. In Atlantic salmon, clear signs of intestinal inflammation, obvious widening of lamina propria and reduced enterocyte vacuolization were observed when soybean meal-based diet—instead of fish meal based one—was fed to the fish [76]. Given the known association of enteric infections with reduced levels of goblet cell response and mucin production [77,78], we speculate that the usage of some commercial feeds might negatively affect the host defence system of the gut. Although no significant difference in mortality was observed among the groups during the trial, it should be noted that keeping conditions in this trial were much more adequate for Asian seabass than those typically maintained at most farms.

Histological analysis of the liver was also performed to detect any potential changes due to different dietary intake. Previous studies on other food fish species showed that dietary intrusion of vegetable oil resulted in swelling of hepatocytes filled with lipid droplets [79,80]. In our study, significantly enlarged hepatocytes filled with lipids droplets were observed in two groups fed with commercial feeds (B&D), but not in Group C. These observations were expected for Groups B and D, as the intrusion of plant-based meal or oil would certainly be present during the production of these commercially compounded feeds. On the other hand, the lack of such phenotypic changes in Group C is surprising, since Feed C had the highest LA and second highest ALA content among the five commercial feeds tested (**Table 2**). A possible explanation for this phenomenon is that during the production of Feed C, anti-inflammatory ingredients, like phosphorus, could have possibly been included, since an independent study found that with the addition of phosphorus in the feed, alterations made to hepatocytes could be improved [81]. Interestingly, these findings tally closely with our liver transcriptomic analysis where only seven DETs were found between Group C and control (Group A), whereas 27 and 127 DETs were found between Group B and Group D against control, respectively (**S4 and S5A Figs**). The severe enlargement of hepatocyte diameter observed within Groups B and D could be an early sign of ‘fatty liver disease’, which is linked to serious implications, such as growth retardation, illness or even death [82], providing a potential explanation for the reduced growth observed when compared to Group C.

## Conclusions

In summary, our study is the first to compare the suitability of three different commercially available pelleted grow-out feeds for juvenile Asian seabass by multiple approaches. Without prior knowledge regarding the ingredient composition of the feeds, the resulting effect of consuming different commercial feeds are significantly reflected in the growth parameters, nutritional value, gut morphology and transcriptomic profile analysed. The effects of the feed compositions used by different manufacturers onto the fish could be further understood by studying the global genes expression profile in greater detail. The expression level of *fads6* and other genes was found to be potentially suitable as a bio-indicator to identify wild-caught Asian seabass from farmed ones. Goblet cell numbers in the mid-gut section of pellet-fed fish were lower than those of controls, indicating potentially negative effects on fish health. By considering the overall effects observed, we would recommend to farmers favouring feeds for the culture of Asian seabass that help to maintain the quality, not just increasing the quantity of the end product (i.e. the fillet).

## Supporting Information

S1 Fig Recording of quantifiable traits and tag number from individual fish based on photographs. (A) The picture of an individual at the beginning at 107dph (weight: 45g). (B) The same fish at the end of experiment at 168 dph (weight: 134g).
(TIF)

S2 Fig Quantification of goblet cell count using different staining methods produced similar results. Midgut sections stained with H/E, goblet cells are large clear vacuoles (A and C); Midgut section stained with PAS staining, goblet cells are stained pink (C); Midgut section stained with Alcian blue staining, goblet cells are stained blue (D). Panel (A-D); 40x magnification, scale bar = 100μm.
(TIF)

S3 Fig Negligible correlation was found between the various histological parameters and body weight (BW). Analysis of potential correlation between different histological parameters of all the samples (36 individuals) and their respective body weight (BW) at 168 dph. Panel A: External circumference of serosa (ECS);, Panel B: Mucosal height (MH); Panel C: Muscularis layer thickness (MLT); and Panel D: Goblet cell number (GCN). Additional abbreviations: R^2^ = coefficient of determination, r = correlation coefficient.
(TIF)

S4 Fig Comparative analysis of microarray-based liver transcriptomic profile of baitfish-fed (Group A) and pellet-fed (Groups B, C and D) Asian seabass. Differentially expressed transcripts (DETs) are defined as P_fdr_ < 0.05 with fold-change cutoff: 2. Significantly expressed transcripts (SETs) are defined as P < 0.05 with no fold-change cutoff. Numerical values represent the amount of DETs or SETs observed when compared between the different groups. Green arrows: up-regulation; red arrows: down-regulation.
(TIF)

S5 Fig Heat map plot of differentially expressed transcripts (DETs) among all the four groups. (A) Thirty-nine DETs were identified through ANOVA analysis. At the level of transcriptome, Group C showed the highest level of similarity to controls (Group A). (B) Four DETs showed the potential to differentiate wild-caught Asian seabass from farmed ones. DETs were defined as P_fdr_ < 0.05 with fold-change cutoff: 2.
(TIF)

S1 Table Nutritional information provided on the packaging labels of the feeds. (XLSX)

S2 Table Fatty acids selected from feeds and fillets for correlation study. (XLSX)

S3 Table Correlation heat map between fatty acids of feeds (A-D) and fillets (A-D). Two clusters of fatty acids were identified that correlated positively within their own cluster, but negatively with fatty acids from the other cluster in both feeds and fillets. Values are represented as Pearson’s correlation efficiency. Green boxes indicate positive correlations, whereas red boxes show negative correlations.
(XLSX)

S4 Table Primer pair sequences, amplicon size of target genes and reference genes used for quantitative real time PCR validation of liver microarray results. (XLSX)

S5 Table Morphometric parameters of the gastrointestinal tract of Asian seabass after consuming frozen fish (A, control) or commercial grow-out feeds (B-D) for 61 days. Results are means ± SEM (n = 9). Morphometric parameters that are significantly different between different feed types as determined by one-way ANOVAs (*—P_adj_ < 0.05). Dunnett’s multiple comparisons test was used to compare between the mean of each group with the mean of the control (group A). Significantly different means are indicated with superscripts (^a^—P_adj_ < 0.05). All values are in μm, except for goblet cell numbers.
(XLSX)

S6 Table Gene Set Enrichment Analysis (GSEA) of KEGG metabolic pathway, using Danio rerio as reference model. Coloured arrows indicated significantly enriched pathways (p <0.05). Direction of arrows indicated up regulated or down regulated.
(XLSX)

S7 Table Microarray validation with real-time qPCR. Values are expression fold-change. Genes that are significantly expressed are noted with (*—P_fdr_ < 0.05).
(XLSX)
