# Supplementary figures and images for: Nutrigenomic and Nutritional Analyses Reveal the Effects of Pelleted Feeds on Asian Seabass (Lates calcarifer)
Source: PLoS One. 2015 Dec 22;10(12):e0145456. doi: 10.1371/journal.pone.0145456 (PMC4687856; doi:10.1371/journal.pone.0145456)

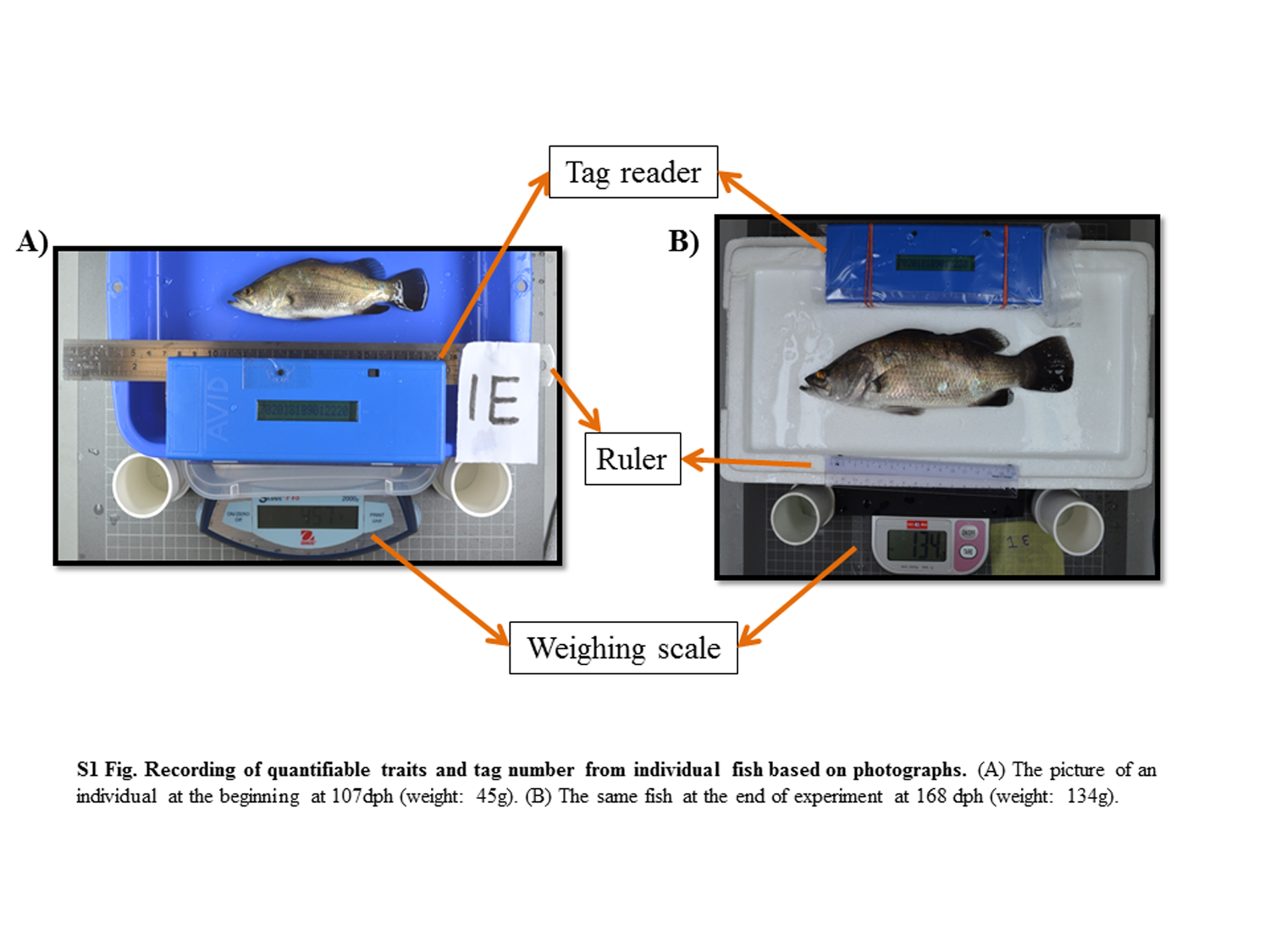

Supplement: S1 Fig — (A) The picture of an individual at the beginning at 107dph (weight: 45g). (B) The same fish at the end of experiment at 168 dph (weight: 134g). (TIF) [file pone.0145456.s001.tif]

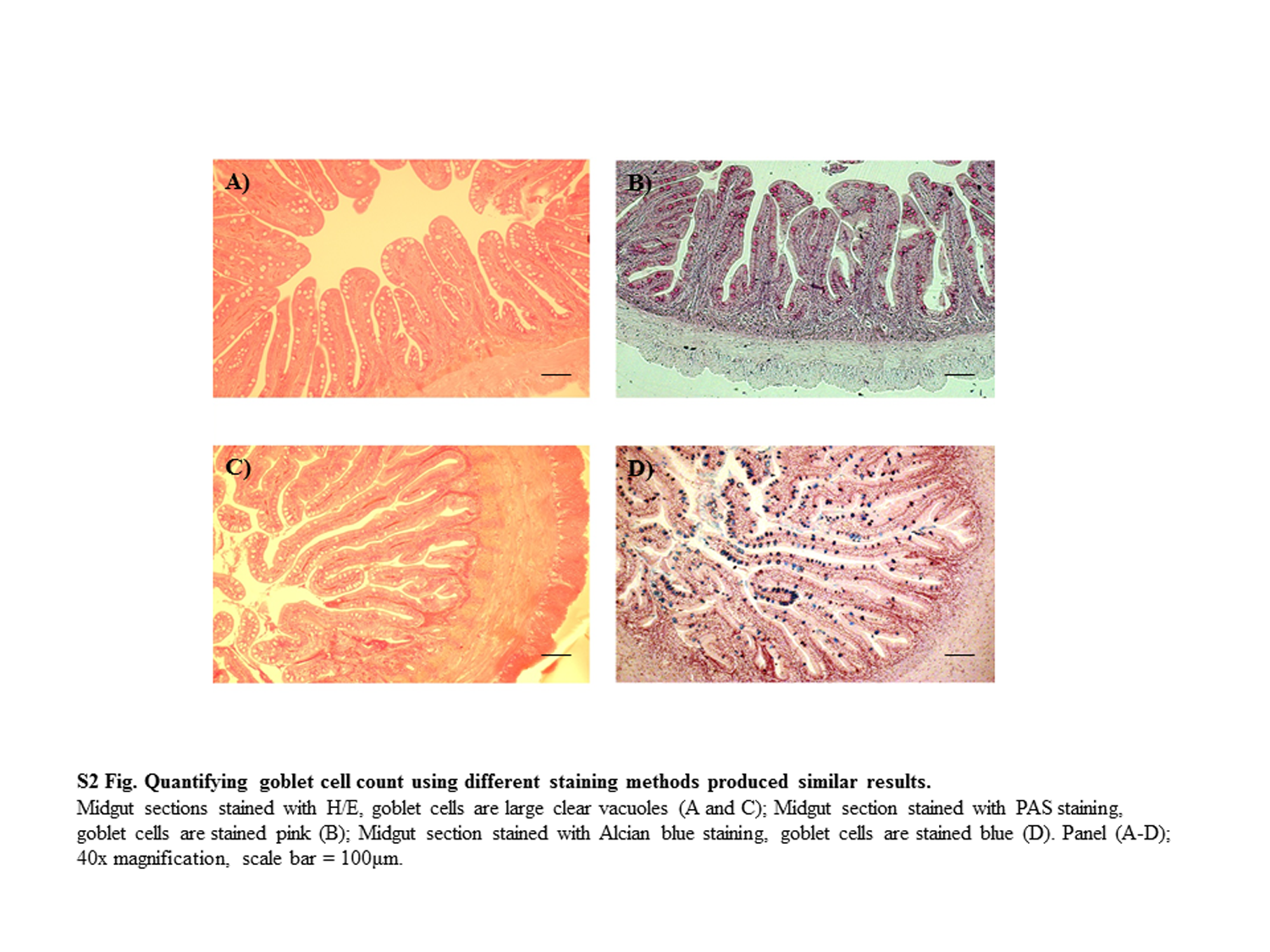

Supplement: S2 Fig — Midgut sections stained with H/E, goblet cells are large clear vacuoles (A and C); Midgut section stained with PAS staining, goblet cells are stained pink (C); Midgut section stained with Alcian blue staining, goblet cells are stained blue (D). Panel (A-D); 40x magnification, scale bar = 100μm. (TIF) [file pone.0145456.s002.tif]

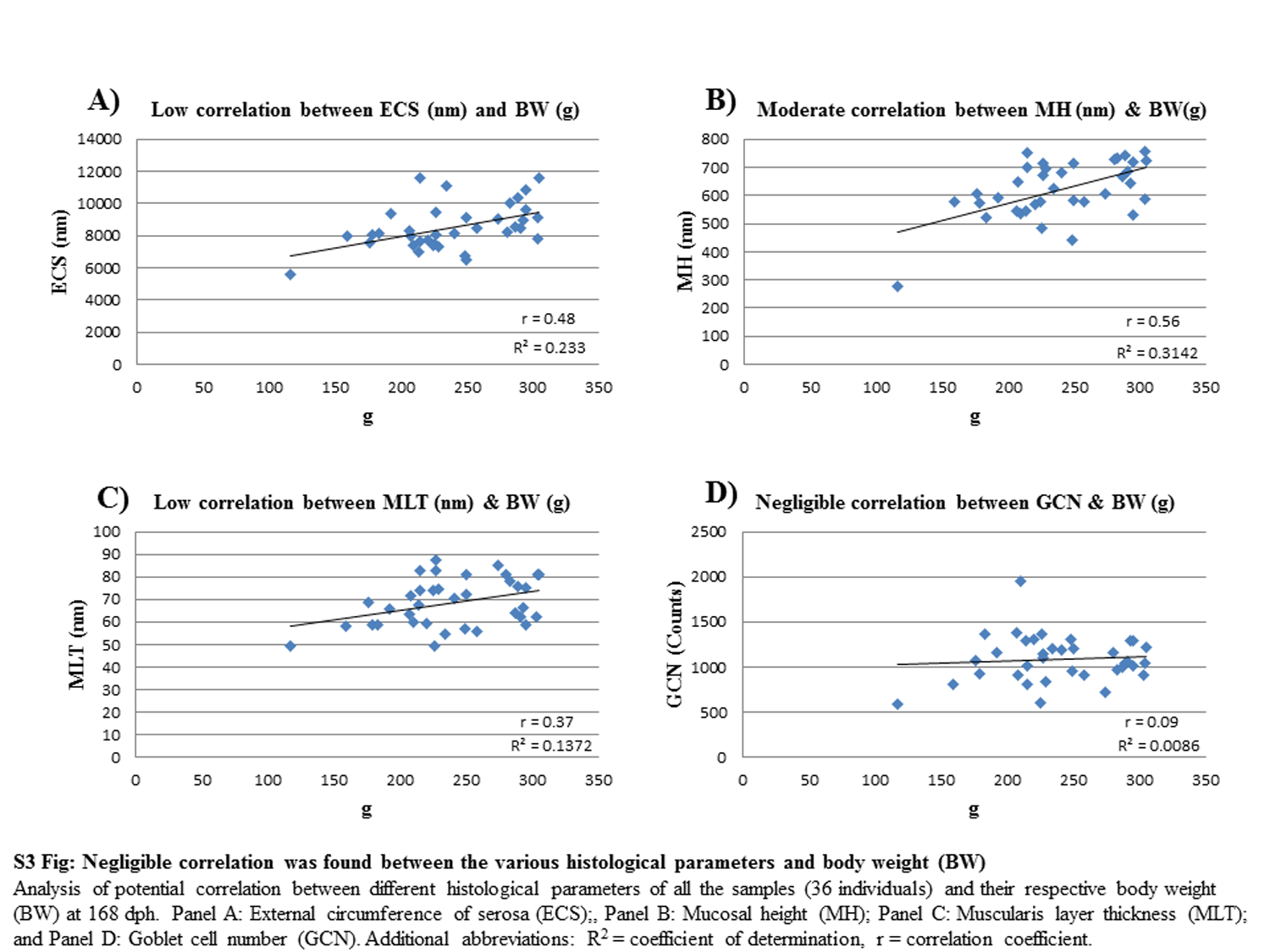

Supplement: S3 Fig — Analysis of potential correlation between different histological parameters of all the samples (36 individuals) and their respective body weight (BW) at 168 dph. Panel A: External circumference of serosa (ECS);, Panel B: Mucosal height (MH); Panel C: Muscularis layer thickness (MLT); and Panel D: Goblet cell number (GCN). Additional abbreviations: R2 = coefficient of determination, r = correlation coefficient. (TIF) [file pone.0145456.s003.tif]

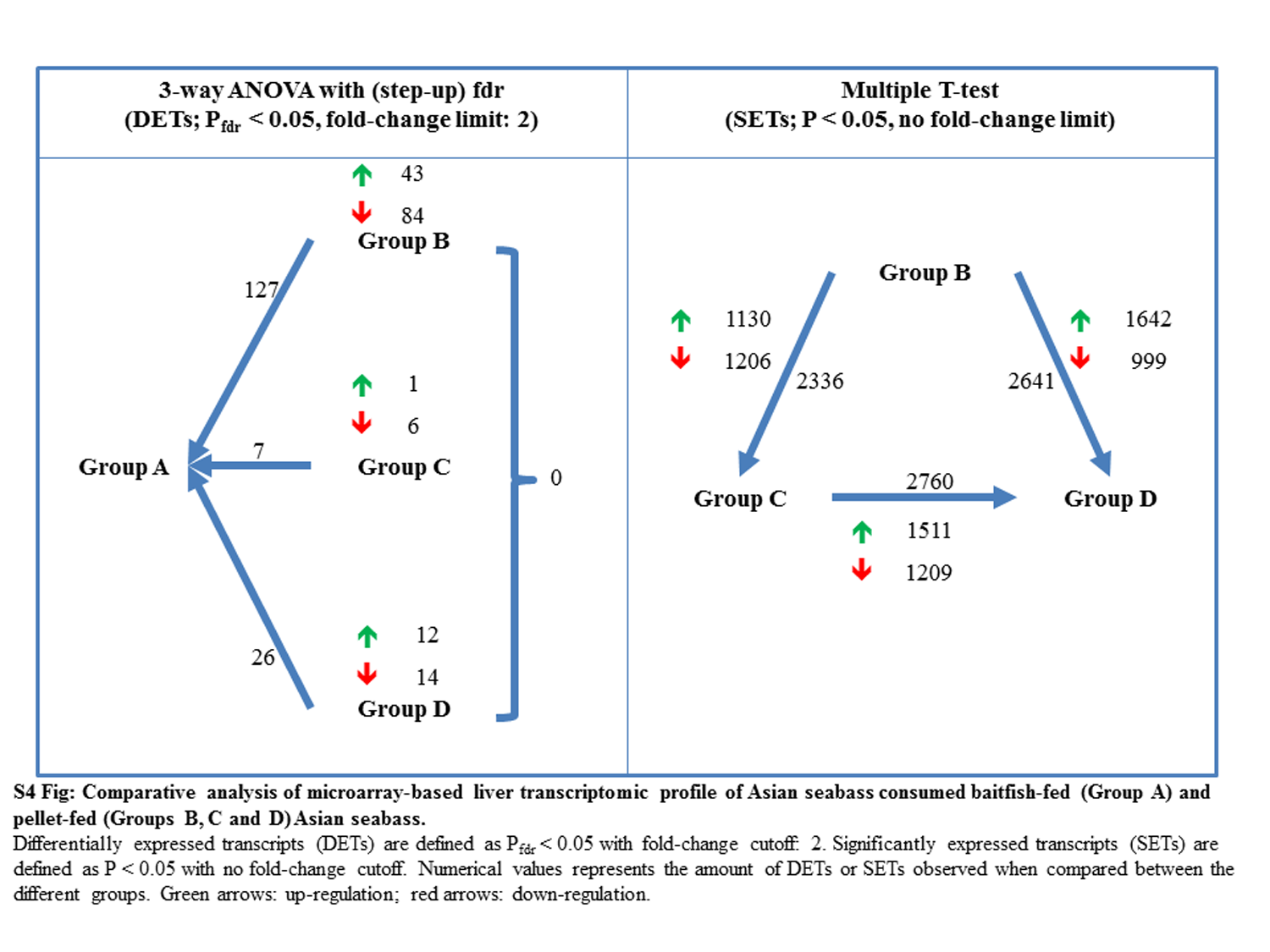

Supplement: S4 Fig — Differentially expressed transcripts (DETs) are defined as Pfdr < 0.05 with fold-change cutoff: 2. Significantly expressed transcripts (SETs) are defined as P < 0.05 with no fold-change cutoff. Numerical values represent the amount of DETs or SETs observed when compared between the different groups. Green arrows: up-regulation; red arrows: down-regulation. (TIF) [file pone.0145456.s004.tif]

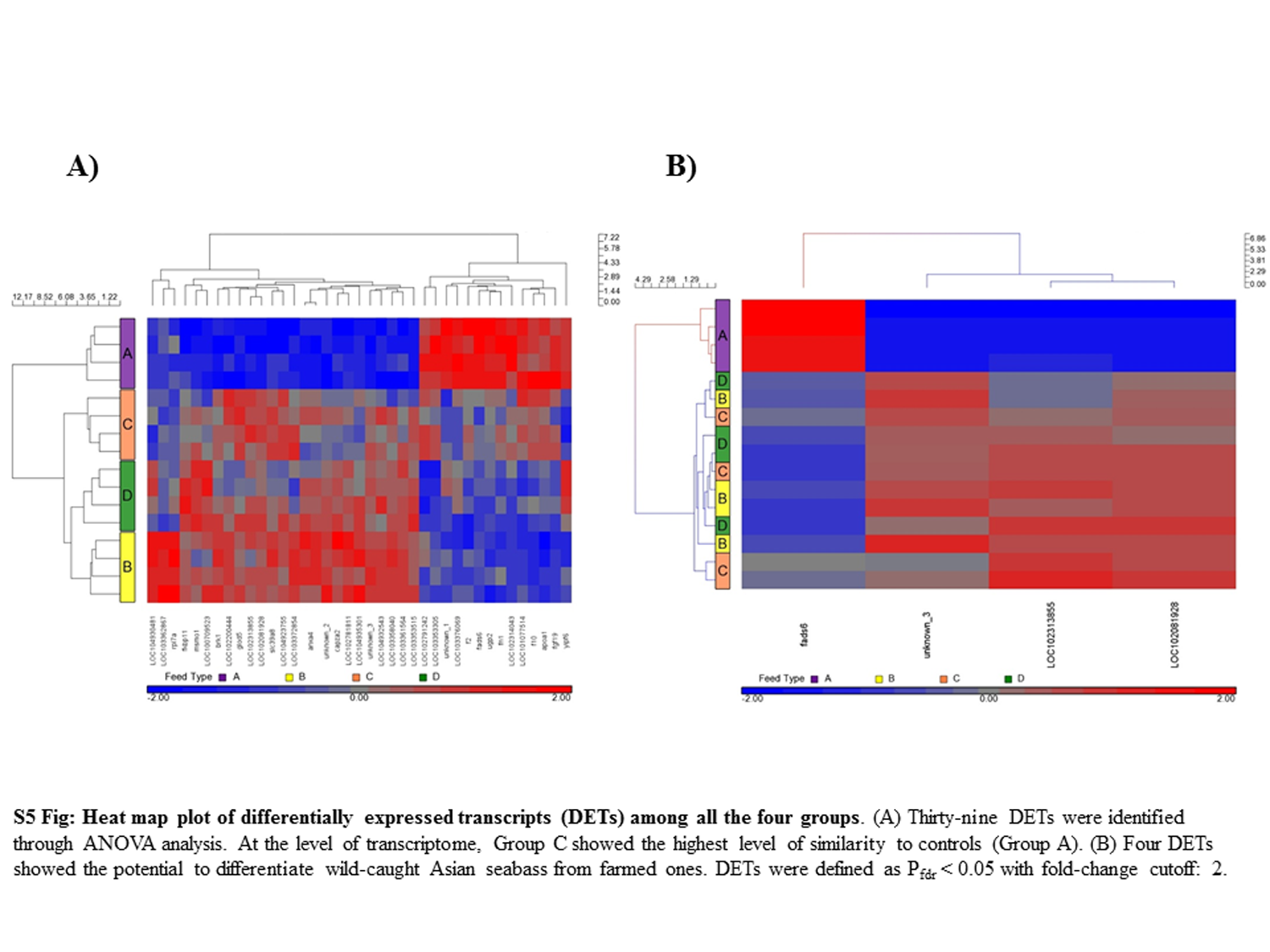

Supplement: S5 Fig — (A) Thirty-nine DETs were identified through ANOVA analysis. At the level of transcriptome, Group C showed the highest level of similarity to controls (Group A). (B) Four DETs showed the potential to differentiate wild-caught Asian seabass from farmed ones. DETs were defined as Pfdr < 0.05 with fold-change cutoff: 2. (TIF) [file pone.0145456.s005.tif]
